# *TP53* mutations in head and neck cancer cells determine the Warburg phenotypic switch creating metabolic vulnerabilities and therapeutic opportunities for stratified therapies

**DOI:** 10.1016/j.canlet.2020.02.032

**Published:** 2020-05-28

**Authors:** Mark D. Wilkie, Emad A. Anaam, Andrew S. Lau, Carlos P. Rubbi, Terence M. Jones, Mark T. Boyd, Nikolina Vlatković

**Affiliations:** a Department of Molecular & Clinical Cancer Medicine, Cancer Research Centre, University of Liverpool, 200 London Road, Liverpool, L3 9TA, UK; b Department of Otorhinolaryngology – Head & Neck Surgery, University Hospital Aintree, Lower Lane, Liverpool, L9 7AL, UK

**Keywords:** p53, Cancer, Glycolysis, Oxidative phosphorylation, Metabolism, Head and neck cancer

## Abstract

Patients with mutated *TP53* have been identified as having comparatively poor outcomes compared to those retaining wild-type p53 in many cancers, including squamous cell carcinomas of the head and neck (SCCHN). We have examined the role of p53 in regulation of metabolism in SCCHN cells and find that loss of p53 function determines the Warburg effect in these cells. Moreover, this metabolic adaptation to loss of p53 function creates an Achilles’ heel for tumour cells that can be exploited for potential therapeutic benefit. Specifically, cells lacking normal wild-type p53 function, whether through mutation or RNAi-mediated downregulation, display a lack of metabolic flexibility, becoming more dependent on glycolysis and losing the ability to increase energy production from oxidative phosphorylation. Thus, cells that have compromised p53 function can be sensitised to ionizing radiation by pre-treatment with a glycolytic inhibitor. These results demonstrate the deterministic role of p53 in regulating energy metabolism and provide proof of principle evidence for an opportunity for patient stratification based on p53 status that can be exploited therapeutically using current standard of care treatment with ionising radiation.

## Highlights

What is already known

• *TP53* mutations are the most common genetic mutation in many cancers, including head and neck cancers.
•Patients with mutated p53 display poor prognosis but no method exists for selectively treating these patients

What this study adds

•We identify and confirm that p53 status (mutant or normal) determines tumour cell metabolic profile
•Metabolic changes induced by loss of p53 function determine sensitivity to metabolic inhibition in combination with the mainstay of current therapy-radiation, and provide an opportunity to increase radiation sensitivity in p53 mutated cells
•The results suggest that a strategy for treating patients with mutations in *TP53* could be readily developed combining the current standard of care (radiotherapy) with short term inhibition of a metabolic pathway (glycolysis).

## 1 Introduction

One of the greatest challenges preventing the successful treatment of cancer derives from difficulties in developing treatment strategies that can distinguish tumour cells from normal and thus provide a substantial therapeutic index. Since cancer is a genetic disease arising from the accumulation of critical somatic mutations, one way to distinguish cancer cells from normal is through the detection of these somatic mutations.

Loss of p53 function is not only the single most common genetic event in cancer, but is also linked with more aggressive disease and poorer patient outcomes in many cancers [1,2]. This is particularly relevant in cancers of the head and neck in which mutations of the *TP53* gene are associated with the worst outcomes [3] and where TCGA has reported a *TP53* mutation frequency of over 80% in the majority of patients who are diagnosed with HPV negative squamous cell carcinomas of the head and neck (SCCHN), making this the single most frequent genetic event in this disease by a large margin [4].

Whilst many therapeutic approaches have been developed that try to take advantage of oncogenic events such as translocations and activation of signalling pathways promoting cell proliferation and survival, loss of tumour suppressor function has proven largely refractory to attempts to target therapeutic interventions [5].

This is not really surprising, since it is conceptually challenging to envisage means to re-activate mutant gene function/s, but fortunately the loss of tumour suppressor gene function in mutant cells frequently creates other functional phenotypic consequences, and these are potentially amenable to targeted intervention. Indeed, loss of p53 function leading, *inter alia*, to loss of induction of genotoxic checkpoint responses is a major component of the effectiveness of many conventional chemotherapeutic agents (reviewed in Ref. [6]). The p53 protein co-ordinates a wide range of cellular processes, primarily through its function as a sequence specific transcriptional regulator [7,8], and recently, it has become clear that p53 has several effects on cellular metabolism. Amongst the first specific clues to this role were generated by studies which identified firstly a novel p53-responsive gene encoded by *C12orf5*, which was subsequently found to encode a fructose-2,6-bisphosphatase with roles in metabolic regulation and apoptosis (hence the gene was named TIGAR, TP53 inducible glycolysis and apoptosis regulator) [9,10].

TIGAR catalyses the removal of phosphate from fructose-2,6-bisphophate generating fructose-6-phosphate, and in so doing promotes gluconeogenesis at the expense of glycolysis whilst also generating a key substrate for the pentose phosphate pathway (PPP). Fructose-2,6-bisphosphate is a potent allosteric activator of phosphofructokinase-1 (PFK-1), which catalyses one of the three major rate limiting steps in glycolysis and the action of TIGAR thus directly suppresses glycolytic flux [10,11]. In addition, studies have demonstrated that TIGAR also suppresses p53-mediated apoptosis, and that this is in part a result of the enzymatic activity of TIGAR as a fructose-2,6-bisphosphatase which results in a reduction in ROS production with one mechanism being the increased PPP flux and increased production of cellular reducing agents [10,12]. Under hypoxic conditions, TIGAR also translocates to mitochondria where it binds to HK2 leading to increased phosphorylation of glucose [13]. The combination of these activities of TIGAR would lead to increased flux through the PPP.

As well as regulating TIGAR, p53 has been shown to have many other impacts on cellular metabolism, ranging from suppressing glucose transporter expression (GLUT-1 and GLUT-4), and promoting oxidative respiration by up-regulating cytochrome *c* oxidase 2 (SCO2) even to having a role in maintaining mitochondrial function and health (reviewed in Ref. [14]).

Given the importance of p53 as a metabolic regulator, and loss of p53 function as both a critical event in carcinogenesis and a determinant of patient disease outcomes, it should hardly be surprising that p53 may provide a key link between carcinogenesis and metabolic adaptations first described over 90 years ago by Warburg, Wind and Negelin [15]. Studies by Myers and colleagues have shown a dependence on glucose as a primary energy source in head and neck cancer cells and comparing HN30 (*TP53* wild-type) and HN31 (*TP53* C176F) cells as well as using RNAi in these lines, they demonstrated that the extent of this dependence was influenced by wild-type p53 expression levels and that glucose dependence was greatest in cells that harboured a *TP53* mutation [16]. Further studies by this group have identified that the metabolic phenotypes of *TP53* wild-type and mutant cells are distinct, confirming the earlier studies of glucose dependence and identifying critical differences in respiration: with mutant cells displaying apparently maximised use of oxidative phosphorylation and wild-type cells retaining significant spare respiratory capacity. These studies also identified a novel therapeutic opportunity based on the glycolytic dependence of the SCCHN cells harbouring mutant *TP53* [17].

A critical issue that arises from these studies is whether p53 inactivation is associated, perhaps indirectly with the regulation of cell metabolism, or whether there is a deterministic consequence of p53 function that causes differential metabolic phenotypes in mutant versus wild-type p53 cells. If the latter, then this might provide for more robust opportunities for developing p53-based stratification of patients for novel therapeutic strategies. To investigate this we have used isogenic cell lines with defined genetically manipulated *TP53* status, including p53 null, wild-type, and various loss of function, dominant negative and gain of function mutants, to examine the role of p53 in SCCHN metabolism and have found that p53 is deterministic in this process. p53 status was further observed to be a predictor of cell metabolism in a panel of (non-isogenic) SCCHN cells that either express wild-type p53, or are null for p53 protein, or express a range of different mutants of p53 (comprising loss of function, dominant negative activity and gain of function). This suggests that p53 status overrides other genetic heterogeneities in conditioning cell metabolism and is therefore a predictor of a clinically significant behaviour of SCCHN. We also find that in absolute terms, loss of p53 function leads to a reduction in respiratory capacity, as well as increasing dependence on glycolysis. Moreover, this leads to increased sensitivity to ionizing radiation (IR is a staple of SCCHN therapy) when combined with inhibition of glycolysis in *TP53* mutant cells, but not in *TP53* wild-type cells. We also show that this is due to increased sensitivity to ROS in *TP53* mutant cells. Thus we propose that since p53 determines this response, future clinical trials stratifying patients based upon *TP53* status, and combining radiotherapy with inhibitors of specific metabolic pathways should be prioritised in SCCHN.

## 2 Materials and methods

### 2.1 Cells and reagents

Parental UM-SCC (University of Michigan Squamous Cell Carcinoma) cell lines (Supplementary Data Table 1), were kindly provided by Prof. Thomas Carey, University of Michigan, MI, USA, and genetically modified derivatives of UM-SCC-1 and UM-SCC-17A lines were kindly provided by Prof. Jeffrey Myers, University of Texas MD Anderson Cancer Centre, TX, USA. Cell lines used include UM-SCC-1 (splice site mutant rendering the cells null for p53 protein expression resulting in loss of p53 function [LOF]) and derivatives of UM-SCC-1 that express either wild-type p53, or a range of p53 missense mutants that display gain of function (GOF); R175H, C176F and R282W, as well as patient derived lines harbouring a range of mutants with different p53 phenotypes: LOF; C242S, dominant negative activity (DNA); V157F, G245C and H193R, and GOF; V157F and (albeit weakly) G245C [[18], [19], [20], [21], [22], [23]]. The identity of all cell lines was confirmed by STR profiling (Powerplex from Promega, Southampton, UK) and comparison with previously published analysis from the Carey group [24]. Isogenic cell lines differing only with respect to p53 expression were routinely monitored by analysis of p53 expression levels as shown in Supplementary Data Fig. 1. Cells were maintained essentially as previously described [25]. Primary antibodies for western blot analysis were: anti-p53 (mouse mAb DO-1, used at 3 μg/ml), anti-MDM2 (mouse mAb IF-2, used at 3 μg/ml), anti-TIGAR (rabbit polyclonal antibody AB10545 used at 1 μg/ml) supplied by Merck-Millipore, Watford, UK, anti-α-vinculin (mouse mAb V9131, used at 1/100,000 dilution) supplied by Sigma-Aldrich, Poole, UK. HRP-conjugated secondary antibodies used were sheep anti-mouse antibody (NA931) at a 1/2500 dilution or donkey anti-rabbit antibody (NA934) at a 1/5000 dilution, both were supplied by GE Healthcare.Where indicated, cells were treated with or exposed to 1 μM staurosporine (LC Laboratories, Woburn, MA, USA), 25 mM 2-deoxy-d-glucose (2-DG), 25 mM N-acetylcysteine (NAC), 100 μM hydrogen peroxide (H_2_O_2_), 2 μM UK5099 all from Sigma-Aldrich, Poole, UK, or ionizing radiation (IR) delivered at room temperature using a CellRad cabinet X-ray cell irradiator (Faxitron Bioptics LLC, Tucson, AZ, USA) calibrated to deliver 2 Gy/min of X-ray radiation.

**Fig. 1:**
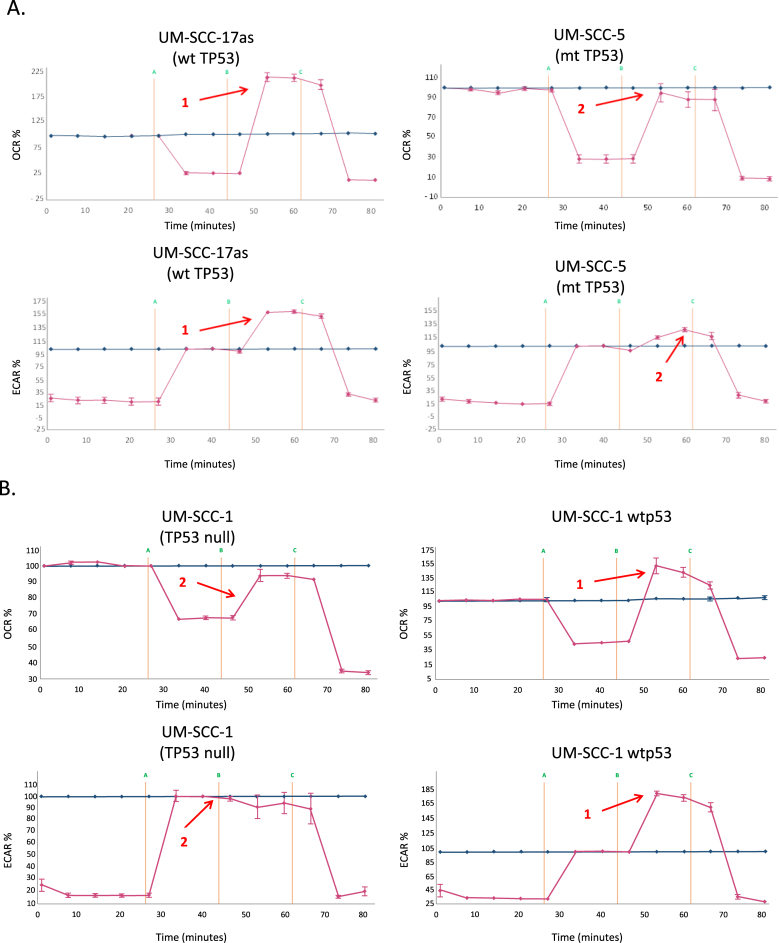

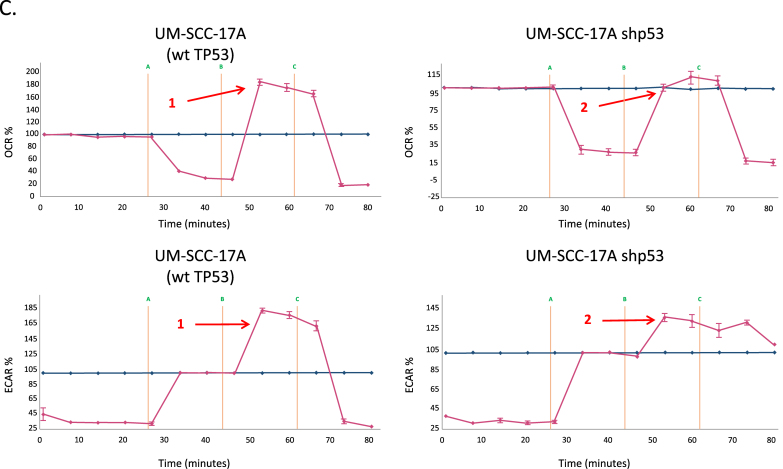
**Wild-type p53 promotes increased oxidative phosphorylation and reduced glycolytic flux in head and neck cancer cells.** A. Relative oxygen consumption rates (OCR) and extracellular acidification rates (ECAR) were measured in mitochondrial and glycolytic stress tests respectively to ascertain levels of oxidative phosphorylation and glycolytic flux using a Seahorse XF24 analyser for representative p53 wild-type (UM-SCC-17as) and mutant (UM-SCC-5) cell lines. Note that similar profiles with respect to p53 status are observed in all lines (Supplementary Data Fig. 2). Data is presented as percentage increases or decreases in OCR and ECAR relative to baseline measurements. The baseline is shown as the blue line on the graphs. B. Experiments were performed as described for A. using p53-null UM-SCC-1 cells and an isogenic derivative that stably expresses wild-type p53 from an integrated lentiviral vector. C. Experiments were performed as described for A. using a wild-type p53 cell line UM-SCC-17A and an isogenic derivative stably expressing shRNA for p53. In each panel arrow 1 indicates increased respiratory capacity (OCR plot) or glycolytic reserve (ECAR plot) associated with wild-type p53 expression (whether endogenous or from a lentiviral vector) in contrast with reduced respiratory capacity and glycolytic reserve highlighted by arrow 2 observed in p53 mutant, p53-null or p53 shRNA-expressing knock-down cells. Results presented are typical from independent experiments performed on at least three occasions and similar data have been obtained by two individuals using both XF24 and XF96 instruments (MDW and EAA). (For interpretation of the references to colour in this figure legend, the reader is referred to the Web version of this article.)

### 2.2 Western blot analysis

Procedures for SDS-PAGE, western blotting and flow cytometric measurement of apoptosis were performed as previously described [[26], [27], [28]]. Briefly, for western blots, protein lysates were resolved by SDS-PAGE prior to transfer to nitrocellulose using a Bio-Rad Trans-Blot Turbo according to the manufacturer's instructions. Blots were processed essentially as described previously [29], probed with the indicated antibodies and signals developed using Clarity™ Western ECL Substrate (#1705060) and detected with a Bio-Rad Chemidoc MP.

### 2.3 Metabolic profile analysis

Metabolic studies were performed using Seahorse XF24 and XF96 analyzers (Seahorse Bioscience, Copenhagen, Denmark) to perform either mitochondrial or glycolytic stress tests, essentially according to the manufacturer's instructions. Experimental readouts are oxygen consumption rate (OCR) in pmol/min and extracellular acidification rate (ECAR) in mpH/min, to provide surrogate measures of oxidative phosphorylation and glycolysis respectively. Data were analysed using Wave software (Seahorse Bioscience, Copenhagen. Denmark). During mitochondrial stress tests, inhibitors were injected sequentially as follows: oligomycin 1.25 μM, FCCP 1.5 μM, and lastly a combination of rotenone and antimycin-A 1 μM. During glycolytic stress tests, inhibitors were injected sequentially as follows: glucose 10 mM, oligomycin 1.25 μM, and finally 2-DG 50 mM. ECAR was measured in mpH/min.

To enable quantification and for comparison of OCR and ECAR metabolic measurements between cell lines, normalisation to sample DNA content was employed. The DNA content of samples following completion of mitochondrial and glycolytic stress tests was determined in Corning® 96-well black bottom plates (Sigma-Aldrich, Poole, UK) and then DNA content was measured using a CyQUANT® cell proliferation assay kit (Invitrogen, Paisley, UK), according to the manufacturer's instructions. Fluorescence was measured at 520 nm following excitation at 508 nm using a POLARstar Omega plate reader (BMG LABTECH, Ortenberg, Germany).

When analysing data between different cell lines non-parametric statistics were used due to the number of samples in each group (<30) as per central limit theorem. Specifically, Mann-Whitney *U* tests were utilised and the significance level for all tests was set at 0.05. For the purposes of meaningful and valid comparison, statistical comparison of absolute values for metabolic parameters was performed only for isogenic cell lines (i.e. UM-SCC-17A versus UM-SCC-17A shp53 and UM-SCC-1 and UM-SCC-1 wtp53), while analysis of relative levels of basal glycolysis and mitochondrial respiration was performed for the remaining cell lines for which wild-type p53 and mutant p53 cell lines were grouped together.

### 2.4 Cell viability and clonogenic survival assays

Cell viability was determined by trypan blue exclusion assays essentially as described [30] and comparisons between treatment conditions performed using Mann-Whitney *U* tests. To study radiation sensitivity, we used clonogenic assays performed as described previously [25,31]. Data were analysed as follows: the plating efficiency (PE) was calculated from the 0 Gy conditions by dividing the number of colonies formed by the number of cells seeded. The number of colonies formed after treatment was then used to calculate the surviving fraction (SF) for each treatment condition, accounting for the plating efficiency: SF = (number of colonies formed/number of cells seeded) × PE. Survival parameters to generate treatment-dose survival curves for treatment conditions were then derived from fitting the data by weighted, stratified, linear regression according to the linear–quadratic formula S(D) = exp(αD+βD [2]), where S is survival following a given dose (D) of IR, which also allowed comparison between treatment conditions as described by Franken et al. [31].

### 2.5 Flow cytometric quantitation of apoptosis and reactive oxygen species

Cells were analysed by flow cytometry using either a FACSCalibur™ instrument in which case data were analysed with CellQuest Pro Software (both from BD Biosciences, Oxford, UK) or with an Attune® acoustic focusing cytometer in which case data were analysed with Attune® Cytometric Software (both from Life Technologies, Paisley, UK). Apoptosis was monitored using Annexin V and propidium iodide staining of cells and measured by flow cytometry essentially as described previously [32].

Reactive oxygen species (ROS) levels were monitored by flow cytometry using the 2′,7′-dichloro-dihydro-fluorescein diacetate (DCFH-DA) assay essentially as described [33].

Again, comparisons between treatment conditions performed using Mann-Whitney *U* tests.

## 3 Results

### 3.1 TP53 status determines metabolic profile in SCCHN cells

We first investigated the metabolic profile of a panel of STR-validated SCCHN cell lines (Supplementary Data Table 1) to determine the relative levels of glycolysis and oxidative phosphorylation/respiration in these cells under standard growth conditions. We measured oxygen consumption rate (OCR) as an indication of respiratory activity and extracellular acidification rate (ECAR) as an indication of glycolysis using extracellular flux analysis. The results of these are shown in Fig. 1 and in Supplementary Data Fig. 2. The data clearly show that cells that retain wild-type p53 (UM-SCC 17as, 17A, and 11A) retain extra respiratory capacity, whereas cells lacking wild-type p53 (UM-SCC-1, 5, 10A, 11B and 81B) operate either at or close to maximal respiratory levels with little or no spare capacity. In addition, we observe in Fig. 1 (and Supplementary Data Fig. 2), that cells harbouring wild-type p53 display greater glycolytic reserves generally than do cells that lack functional p53 (UM-SCC 1, 5, 11B, 81B and to a lesser extent 10A [which display a glycolytic reserve of ~30%]). Curiously the mutant cell line that retains some unused glycolytic reserve (UM-SCC-10A) also fails to completely respond to the inhibition of glycolysis leading to a reduction in ECAR by the addition of the competitive inhibitor 2-deoxy-glucose (UM-SCC-10A, Supplementary data Fig. 2B). The basis for this remains unclear, but subsequent studies have identified a role for p53 in this phenotype that warrants further investigation (see below in discussion of Fig. 1C), and we have observed that there is a significant (*P* < 0.01) increase in non-glycolytic acidification in mutant vs wild-type cell lines as illustrated in Supplementary Data Fig. 3 B.

**Fig. 2:**
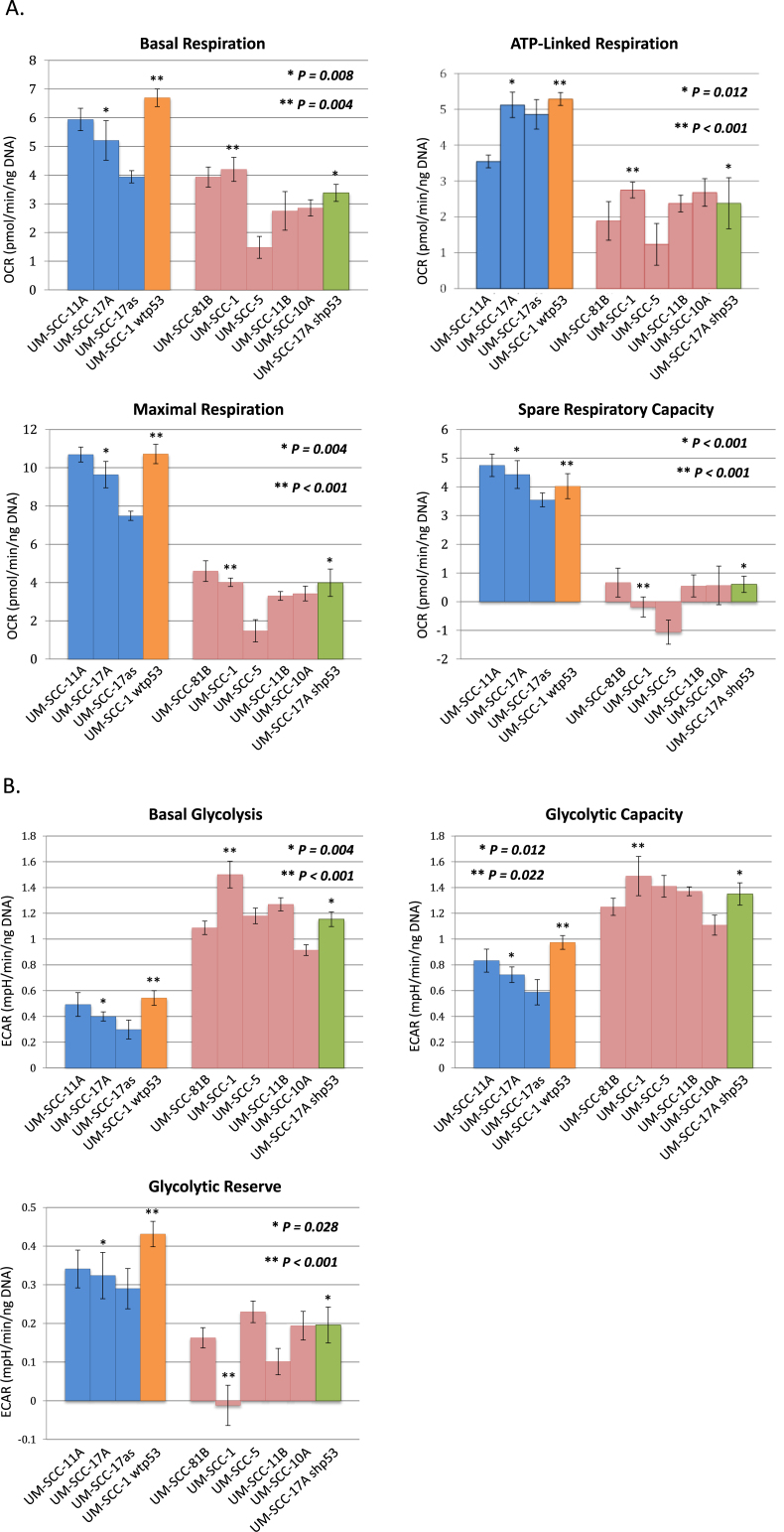

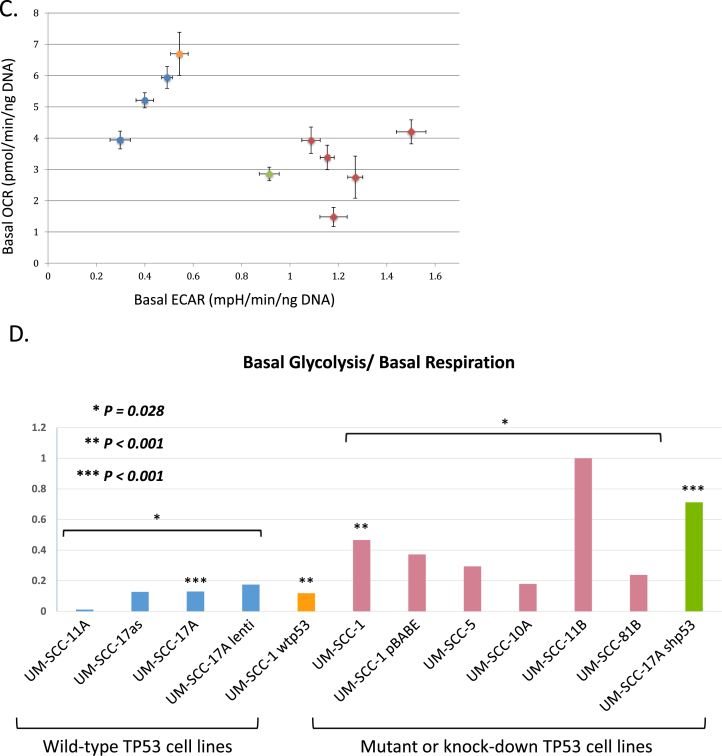
**Metabolic profiling of head and neck cancer cells can dichotomise cells according to p53 status.** Quantitative OCR and ECAR data, normalised to DNA content for cells expressing wild-type p53 (blue), and mutant cells (red). Orange bars represent data from a p53 null cell line manipulated to express wild-type p53 and green bars derive from a p53 knock-down wild-type cell line. As stated in the Methods, statistical comparison of absolute values for metabolic parameters was performed for isogenic cell lines (i.e. UM-SCC-17A versus UM-SCC-17A shp53 and UM-SCC-1 and UM-SCC-1 wtp53), while analysis of relative levels of basal glycolysis and mitochondrial respiration was performed for the remaining cell lines for which wild-type p53 and mutant p53 cell lines were assessed as groups as presented. A. displays data from mitochondrial stress tests with statistical analysis as follows: basal respiration * *P* = 0.008 and **P = 0.004; ATP-linked respiration * *P* = 0.012 and **P < 0.001; maximal respiration * *P* = 0.004 and **P < 0.001; spare respiratory capacity * *P* < 0.001 and **P < 0.001. B. displays data from glycolytic stress tests with statistical analysis as follows: basal glycolysis * *P* = 0.004 and **P < 0.001; glycolytic capacity * *P* = 0.012 and **P = 0.022; glycolytic reserve * *P* = 0.028 and **P < 0.001. C. Plot of basal ECAR versus OCR dichotomises samples into p53 wild-type and p53 compromised (mutant/null/knock-down) groupings. D. Additional studies performed using a Seahorse XF96 analyser and plotting basal ECAR/OCR as a ratio indicative of the Warburg effect: higher values are typical of normal cells with greater use of oxidative phosphorylation and lower values are typical of loss of normal p53 function, indicative of increased dependence on glycolysis. Grouped cell lines harbouring mutant p53 demonstrated a significantly higher ratio of basal glycolysis to basal respiration (*P = 0.028). Direct comparison of the isogenic cell lines showed a similar pattern (**P < 0.001 and ***P < 0.001). UM-SCC-17A lenti, is a UM-SCC-17A derivative that contains a lentiviral vector, UM-SCC-1 wtp53, is a UM-SCC-1 derivative that expresses a wtp53 cDNA, UM-SCC-1 pBABE is a UM-SCC-1 derivative that contains the pBABE retroviral vector, and UM-SCC-17A shp53, is a UM-SCC-17A derivative that contains a lentiviral vector expressing a shRNA for p53. Results presented are typical from independent experiments performed on at least three occasions and similar data have been obtained by two individuals using both XF24 and XF96 instruments (MDW and EAA). (For interpretation of the references to colour in this figure legend, the reader is referred to the Web version of this article.)

**Fig. 3:**
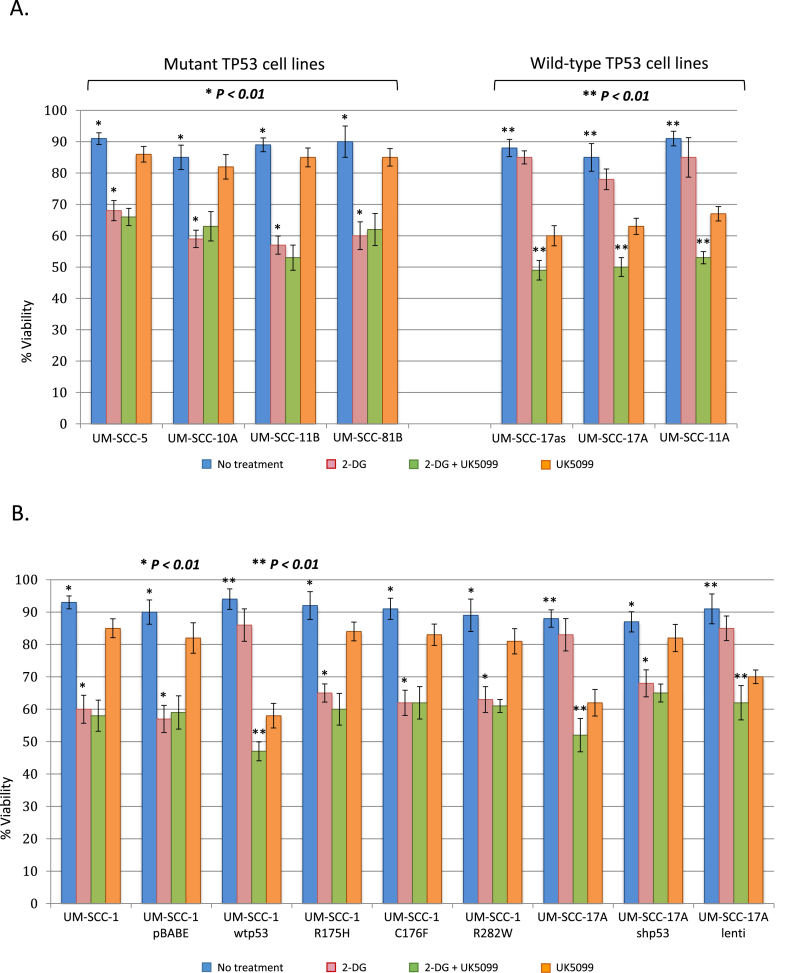
**Head and neck cancer cells with mutant TP53 display increased sensitivity to glycolytic inhibition with 2-DG.** Viability of cells treated with the indicated compounds was measured by trypan blue exclusion. A. and B. The indicated cell lines were exposed to either a no treatment vehicle control, 2-DG, UK5099 or a combination of 2-G + UK50099, as indicated, for 48 h prior to staining. Values represent the mean from three separate experiments and error bars represent the standard error of the mean. In all cell lines with abrogation of wtp53 function (either through mutation or knock-down) addition of 2-DG alone significantly reduced viability (*P < 0.01 in all cases), while in cell lines harbouring functional wtp53 both 2-DG and UK5099 were required to significantly reduced viability (**P < 0.01 in all cases). (For interpretation of the references to colour in this figure legend, the reader is referred to the Web version of this article.)

To further investigate the association between p53 status (nonsense or missense mutant versus wild-type) and metabolic capacity/profile, we next set out to examine whether the association of the observed metabolic phenotypes with p53 status is due to a deterministic function of p53.

As shown in Fig. 1B and C, functional studies identify that reintroducing wild-type p53 into a p53-null cell line (UM-SCC-1) or compromising wild-type p53 function in a wild type p53 cell line (UM-SCC-17A), leads to a predictable response that accords with the association already observed. Specifically, Fig. 1B shows that in isogenic derivatives of p53-null UM-SCC-1 that harbour wild-type p53 stably expressed from a lentiviral vector [16,34], both OCR and ECAR are transformed in a dominant fashion that resembles the wild-type p53 cell line profiles shown in Fig. 1A and in Supplementary Data Fig. 2. Similarly, using RNAi to suppress wild-type p53 expression in UM-SCC 17A cells, results in the reduced spare respiratory capacity and reduced glycolytic reserve observed in p53 mutant cell lines (compare Fig. 1B with data shown in Fig. 1A and B and Supplementary Data Fig. 2). Since manipulation of p53 robustly determines metabolic profile, we therefore conclude that p53 can determine the metabolic profile in SCCHN cells and that loss of p53 function leads to a Warburg type profile displaying greater apparent dependence on glycolysis and reduced respiratory capacity [15,35,36].

### 3.2 p53 suppresses glycolysis and promotes increased respiration

p53 status might impact upon metabolism in several ways, and whilst the studies depicted in Fig. 1 and Supplementary Data Fig. 2 indicate a role for p53 in regulating the relative levels of glycolysis and respiration, we wanted to determine whether the relative changes in capacity are due to an actual increase or decrease in the cells capacity for glycolysis or oxidative phosphorylation, or due to changes in both. To achieve this we used sample normalisation by DNA content to perform similar experiments in a more quantitative manner. As Fig. 2A illustrates, basal respiration, ATP-linked respiration and maximal respiration are all lower in p53 mutant cells cf. wild-type cells. Moreover, reverse genetic experiments in isogenic cell lines in which wild-type p53 is introduced (UM-SCC 1 wt p53) or suppressed (UM-SCC 17A shp53) mirror this phenotype.

Since mutant p53 cell lines have a lower respiratory activity, and since respiration is a more energetically efficient process than glycolysis, one might anticipate that the cells display such a limited respiratory activity because they are operating at close to their maximal available respiratory capacity. As Fig. 2A shows, this is indeed the case. In contrast, p53 wild-type cells display substantial spare respiratory capacity as well as higher absolute levels of respiration. In Fig. 2B, we can see that both the absolute basal levels of glycolysis and total glycolytic capacity of these cells are substantially higher in SCCHN cells that have compromised p53 function (harbouring variously null, mutant or a knock-down of p53) than in p53-wild-type cells. These p53-compromised cells also display greatly reduced glycolytic reserve capacity, and as Fig. 2C shows, combining analysis of measurements of glycolysis and of respiratory activity provides a means to dichotomise cells based upon their metabolic activity that ultimately also predicts their *TP53* status. In further experiments shown in Fig. 2D the “Warburg effect” or phenotype is indicated by ascribing to each cell line a value derived from the ratio of basal glycolysis/basal respiration (raw data for these experiments are presented in Supplementary Data Fig. 3). This results in clear dichotomisation of cells into p53 wild-type and p53 compromised groups.

### 3.3 Loss of TP53 function leads to increased glycolytic dependence and a Warburg phenotype

Following from the results presented in Fig. 2, it would be expected that SCCHN cells in which p53 is compromised will be more dependent upon glycolysis, and due to their reduced spare glycolytic capacity, will therefore be more sensitive to inhibition of glycolysis than p53 wild-type cells. If this was found to be correct, then therapeutic strategies could be envisaged that would take advantage of the anticipated metabolic sensitivity created by this oncogenic adaptation. To examine whether p53-compromised cells do indeed have a greater dependence on glycolysis, we measured the viability of cells in the presence and absence of the glycolysis inhibitor 2-deoxy-d-glucose (2-DG) and the mitochondrial pyruvate carrier (MPC) inhibitor UK5099, which directly inhibits the TCA cycle and thus ultimately deprives the electron transport chain of essential intermediates [37]. Fig. 3 demonstrates that cells harbouring mutant p53 are sensitive to 2-DG alone, but not to UK5099 when used as solo agents, whereas p53 wild-type cells display little sensitivity to inhibition of glycolysis alone with 2-DG, but are, as expected, more sensitive to UK5099. In both mutant and wild-type cells the combination of 2-DG with UK5099 displayed little or no further impact on viability as might be expected since in each class of cells one or other inhibitor is targeting the primary energy generating pathway, glycolysis in the mutant cells and respiration in p53-wild-type cells. Critically, in UM-SCC 1 cells (p53 null), that express wild-type p53 (UM-SCC 1 wtp53), the inhibition of viability induced by treatment with 2-DG is lost, and the cells also become sensitive to UK5099, thus indicating the deterministic nature of p53 expression upon these phenotypes (Fig. 3). Conversely, down-regulation of wild-type p53 by RNAi (UM-SCC 17A shp53), leads to loss of sensitivity to UK5099 and increased sensitivity to 2-DG compared to parental p53 wild-type UM-SCC 17A cells (Fig. 3A).

It is well documented that not all *TP53* mutations have the same functional or phenotypic impacts [3,34,38]. Therefore we examined the impact of inhibiting glycolysis or respiration in isogenic derivatives of the UM-SCC 1 line that express different mutations of *TP53* which occur frequently in SCCHN, two structural mutants R175H and C176F and a contact mutant R282W [39]. Results presented in Fig. 3B show that expression of any of these p53 mutants results in comparable metabolic dependencies to the parental p53 null UM-SCC 1 line in contrast to the same cells expressing wild-type p53 (UM-SCC 1 wtp53).

These results demonstrate that expression of wild-type p53 determines the balance of the metabolic processes in SCCHN cells leading to increased dependence on respiration and less dependence on glycolysis and thus loss of p53 function through mutation or down-regulation promotes a Warburg phenotype.

### 3.4 Inhibition of glycolytic flux radiosensitises SCCHN cells that harbour mutant, but not wild-type p53

**Fig. 4:**
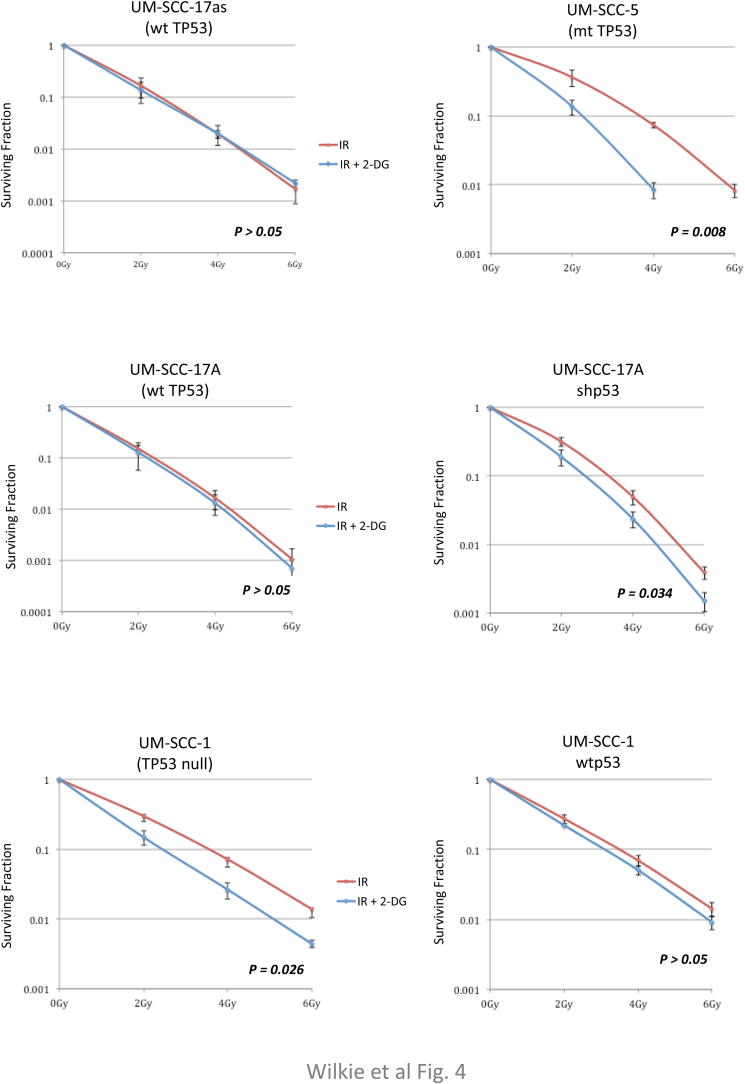
**Head and neck cancer cells harbouring mutant p53 or with knocked-down p53 are sensitised to ionizing radiation by inhibition of glycolysis.** Clonogenic assays demonstrate that the presence of wild-type p53 (endogenous or expressed from an integrated lentiviral vector) suppresses the increased radio-sensitivity induced by inhibition of glycolysis. Cell lines, as indicated, were plated and the next day were treated with vehicle or with the glycolytic inhibitor 2-DG for one hour prior to exposure to the indicated radiation dose. Survival data were fitted to the standard linear quadratic equation S(D) = exp(αD+βD [2]) as described previously [25]. Results represent mean values obtained from at least three separate experiments and error bars represent the standard error of the mean. *P* values were calculated according to the method described by Franken [31].

External beam radiation therapy (EBRT) is a mainstay of treatment for SCCHNs either used as a primary or adjuvant treatment modality alone or in combination with chemotherapy [40,41]. Cell killing by radiation is essentially an oxygen-dependent process [42,43] and one of the major by-products of respiration are a variety of reactive oxygen species (ROS). Because of the importance of p53 mutations and loss of p53 function being linked with poor outcomes in SCCHN [3], we considered the possibility that inhibiting glycolysis would potentiate radiation sensitivity in p53 mutant cells as a result of their increased dependence on glycolysis. As shown in Fig. 4A, addition of 2-DG to cells 1 h prior to irradiation significantly potentiates the suppression of viable colony growth in p53 mutant cell lines, but not in less glycolysis-dependent p53-wild-type cells, as anticipated from the above metabolic studies. The data presented in Fig. 4B again confirm the deterministic nature of p53 in these responses being based on isogenic cell lines in which wild-type p53 is either down-regulated (UM-SCC 17A shp53) or re-introduced into a p53 null background (UM-SCC 1 wtp53). Whilst 2-DG is primarily an inhibitor of glycolysis, it is well documented to have additional effects that are not due to inhibition of glucose catabolism [44]. As with many drugs, whilst the mechanism of action may be unclear (metformin is a good example of this), the therapeutic benefits remain undisputed and in the case of 2-DG there remain good arguments for using it as a part of a potential cancer therapy, particular as a chemo-radiation combination approach [45]. We have performed similar studies of the radiation sensitising effect of another inhibitor of glycolysis (3-bromopyruvate [3-BP]) and have obtained similar results as Supplementary Data Fig. 5 demonstrates. Again, there are concerns regarding the specificity of 3-BP, it is certainly an alkylating agent with a number of substrates, nevertheless, it is also clearly an inhibitor of glycolysis [46].

**Fig. 5:**
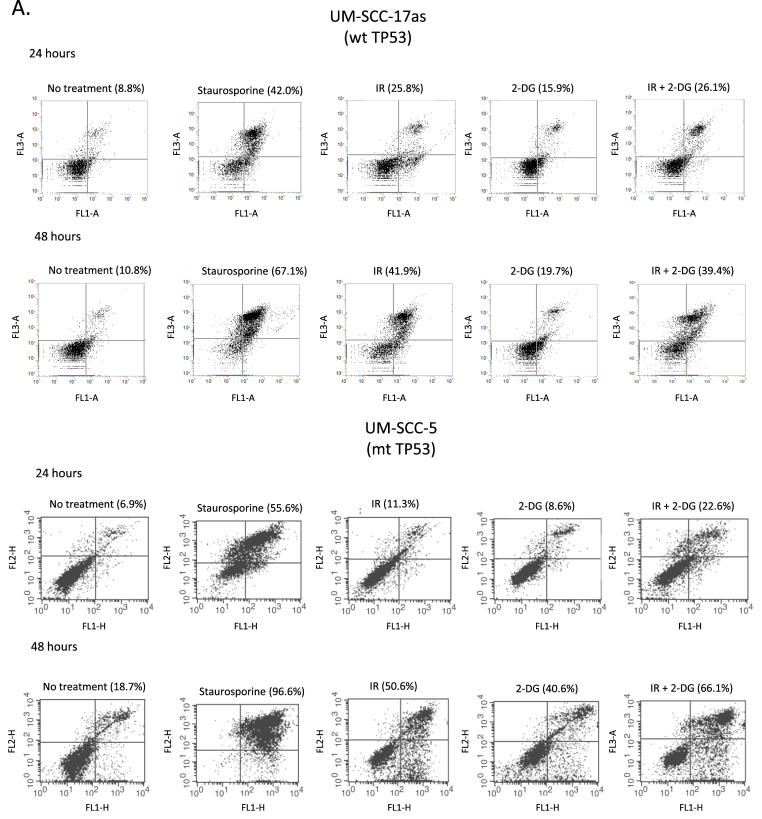

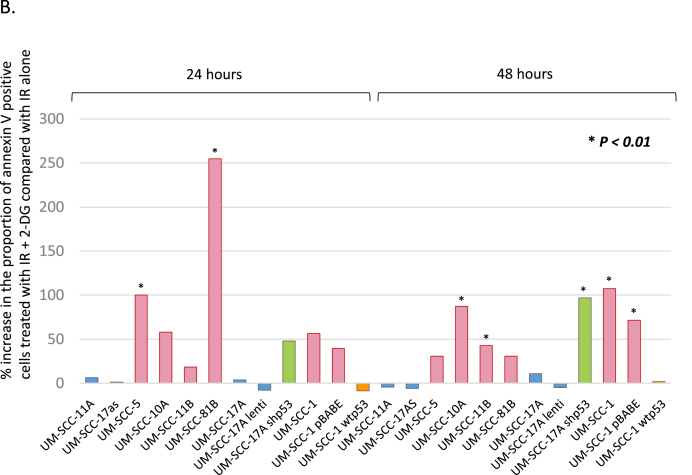
**The radiosensitisation of head and neck cancer cells with compromised p53 function correlates with radiation-induced apoptosis**. A. Flow cytometry analysis of representative p53 wild-type (UM-SCC-17as) and mutant (UM-SCC-5) cell lines treated as indicated with either no treatment, staurosporine as a positive control for detection of apoptosis induction, ionising radiation (IR), 2-DG or a combination of IR plus 2-DG harvested 24 or 48 h later as indicated. In all plots the abscissa indicates Annexin V detection and the ordinate is propidium iodide detection. Percentages represent Annexin V positivity. B. Summarises the results for similar experiments to those described in A. performed on the indicated cell lines. As in Fig. 2, blue bars represent data from cell lines with wild-type p53, red bars represent data from cell lines that are either p53 null or harbouring mutant p53, orange bars represent data from a p53 null cell line manipulated to express wild-type p53 and green bars derive from a p53 knock-down wild-type cell line. In all cell lines with abrogation of wtp53 function (either through mutation or knock-down) addition of 2-DG to IR significantly increased the proportion of annexin V positive cells at either 24 or 48 h (*P < 0.01 in all cases), while in cell lines harbouring functional wtp53 no significant increase was observed. Results presented are typical results from an independent experiment that has been repeated on at least three occasions. (For interpretation of the references to colour in this figure legend, the reader is referred to the Web version of this article.)

These results suggest that inhibition of glycolysis could be used to augment the effectiveness of radiation for patients with tumours harbouring p53 mutations. In Fig. 5A, we show examples of flow cytometric analysis of apoptosis using Annexin V/propidium iodide staining in a wild-type p53 (UM-SCC 17as) and a mutant p53 line (UM-SCC-5) which shows that IR+2-DG increases apoptosis in p53 mutant but not in p53-wild-type cells. Further support for this is summarised in Fig. 5B which provides additional evidence that p53 has a deterministic role in this process in isogenic cell lines (see also Supplementary Data Fig. 4 and Supplementary Data Tables 2 and 3). As might be anticipated from the data shown in Figs. 4 and 5B, p53 status also distinguishes cellular responses to radiation-induced apoptosis with p53 mutant cells displaying the highest increases in apoptosis resulting from the addition of 2-DG treatment prior to exposure to IR and wild-type displaying little or no additional apoptosis when glycolysis is inhibited with 2-DG prior to IR. These results are reminiscent of the earlier ECAR and OCR dichotomisation and place the same samples into two identical groupings as shown in Fig. 2C and D. An important additional point that arises from these data relates to the toxicity of 2-DG. Analysis of apoptosis using Annexin V indicates that exposure of these cells to 2-DG alone, used at 25 mM (for all experiments), and applied for 24 h for these experiments, in contrast to the 1 h treatment used for the clonogenic assay experiments, increases Annexin V positivity by an average of 3–4% (3.74 ± 0.9% for wild type and 3.1 ± 0.6% for mutant cell lines [mean ± SEM]) in the absence of radiation in both wild-type and p53 mutant cells (see Fig. 5 and Supplementary Fig. 4A–C). Thus we anticipate that exposure to 2-DG for an hour or less is likely to have little effect on cell viability in the absence of radiation.

### 3.5 Radiosensitisation of SCCHN cells harbouring mutant TP53 is mediated by ROS

**Fig. 6:**
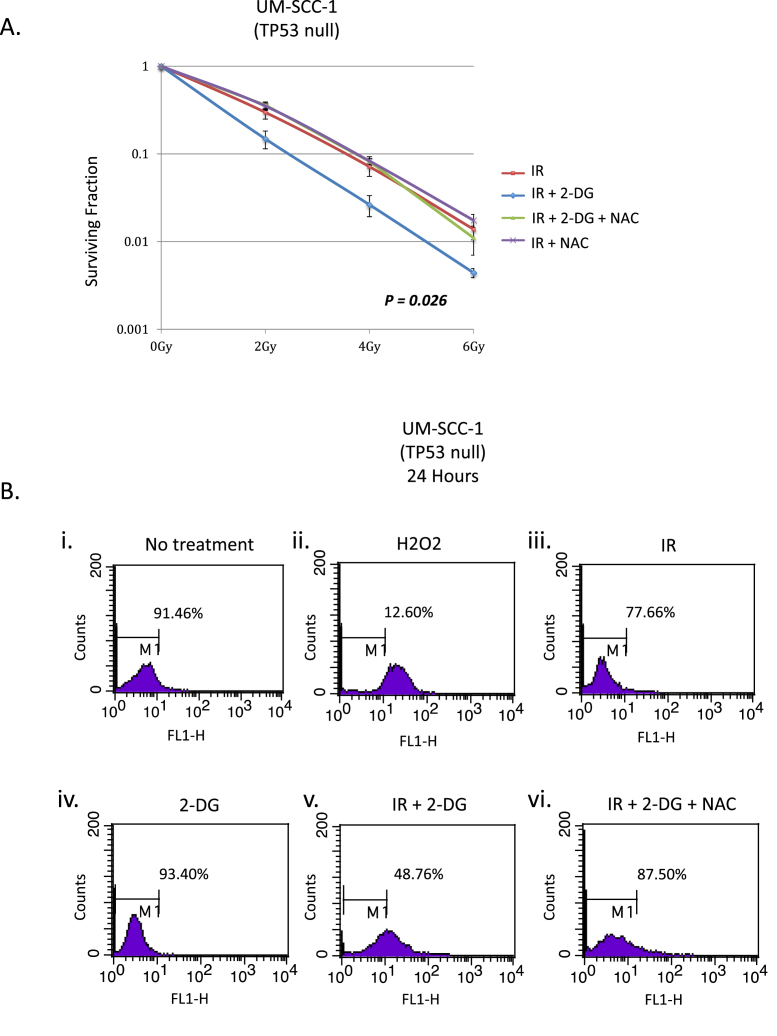
**Radiosensitisation in p53 mutant cells is due to production of reactive oxygen species (ROS).** A. Data from a clonogenic assay demonstrating that the radiosensitisation induced by inhibiting glycolysis can be rescued by adding N-acetylcysteine which acts to increase the levels of reduced glutathione. Results represent mean values obtained from at least three separate experiments and error bars represent the standard error of the mean. *P* values were calculated according to the method described by Franken [31]. B. Flow cytometry analysis of cells either untreated, or exposed to hydrogen peroxide, ionising radiation (IR), 2-DG or the indicated combinations and harvested 24 h later. Percentages indicate the percent of events (cells) within the gated region, indicating the majority of the normal ROS level. ROS is measured in the FL1-H channel and is quantitated using 2′,7′-dichloro-dihydro-fluorescein diacetate. See also Supplementary Data Tables 4 and 5 for additional flow cytometry data. In all cell lines with abrogation of wtp53 function (either through mutation or knock-down) addition of 2-DG to IR resulted in a significant shift in fluorescence indicative of increased ROS (measured by the proportion of cells inside/outside the gated parameter) (*P < 0.05 in all cases), while in cell lines harbouring functional wtp53 no significant increase was observed. Results presented are typical results from an independent experiment that has been repeated on at least three occasions.

Since inhibition of glycolysis rendered p53 mutant cells more sensitive to radiation, and since radiation acts in an oxygen-dependent manner to kill cells, we therefore investigated the role of ROS in this process. Specifically, we investigated whether the increased radiation sensitivity displayed by p53 mutant SCCHN cells treated with 2-DG to inhibit glycolysis could be abrogated by adding N-acetyl cysteine (NAC) to augment cellular reducing capacity [47]. Fig. 6A demonstrates that adding NAC completely rescues the increase in radiation sensitivity induced in p53 mutant cells by 2-DG. To further investigate the relevance of ROS in mediating the observed effects, we used a flow cytometric assay to quantitate ROS in cells under similar conditions to those used in Fig. 6A. Fig. 6B shows that adding NAC to cells that had been irradiated and treated with 2-DG (panel vi) results in a dramatic decrease in the level of ROS detected, returning cells to levels of ROS that are comparable to those detected in untreated cells (panel i). Similar results were obtained for all p53 mutant cell lines as summarised in Supplementary Data Tables 4 and 5. Note that in Fig. 6B panel iv, there is no increase in ROS production in the presence of 2-DG alone, thus cells are not producing more ROS, but the rescue of IR sensitisation with NAC, shows that ROS that is produced is more cytotoxic. This suggests that the increased radiotoxicity in the presence of 2-DG is due to a reduction in the capacity of the cell to neutralise ROS that are produced. The rescue of this effect with NAC supports this conclusion.

We conclude that the increased sensitivity to IR resulting from the inhibition of glycolysis with 2-DG in p53 mutant cells is due to the reduced capacity of mutant cells to tolerate ROS and not a consequence of increased production of ROS when cells are prevented from utilising glycolysis as an major source of energy.

## 4 Discussion

Previous studies in a number of different types of cancer cells have identified a role for p53 as a regulator of a range of cellular processes directly and indirectly contributing to the regulation of many different metabolic pathways [48]. In head and neck cancer cells, a series of excellent studies from the Myers’ group have shown that cells expressing lower levels of p53 protein displayed reduced metabolic diversity, with an increased dependence on glucose which can form the basis for a therapeutic strategy [49]. These studies have raised several critical questions, not least of which are: i) To what extent does *TP53* status determine metabolic changes in SCCHN cells, for example would re-introduction of wild-type p53 re-establish metabolic flexibility and ii) in a more quantitative sense, what is the impact of *TP53* status on the absolute, rather than relative, levels of respiratory and glycolytic metabolism? The present study provides answers to both of these questions. Our data demonstrate that re-introduction of wild-type p53 can reverse the metabolic adaptation of p53 null mutant cells (but note that this does not mean that re-introducing wild-type p53 into cells expressing dominant negative or gain of function mutants of p53 would have the same effect). We also find that not only does the metabolic profile, i.e. the relative levels of glycolysis and respiration change away from respiration towards glycolysis in cells that lack p53 activity, but also the capacity for respiration is substantially reduced and at the same time, mutant p53 cells display an increase in glycolytic capacity. Given the relative inefficiency of glycolysis compared to respiration in generating energy from glucose, this increase in glycolytic capacity is not sufficient to compensate for the reduction in respiratory activity and so there must be important adaptive advantages to the cells to drive such an energetically negative alteration. One explanation is the need for increased flux through the pentose phosphate pathway to increase reducing agents and to produce the NADPH required for macromolecular biosynthesis and promote cell survival in cells stressed by oncogenic events [50]. Regardless of the answer to such questions relating to the pressures that tumour cells experience and adapt to, our data indicate that the metabolic balance and activity of individual metabolic processes is determined by the *TP53* status of cells.

Because of the critical role of radiation therapy in head and neck cancer treatment, we have also investigated the impact of *TP53* status on short-term and long-term cellular radiation responses (apoptosis and clonogenicity respectively), and again find that there are distinct differences that are determined by p53. Due to the decreased capacity of p53 mutant cells for respiration, mutant cells depend more on glycolysis for energy production. We have found that this renders these cells more sensitive to IR in the presence of inhibitors of glycolysis such as 2-DG. Our results suggest that this is due to decrease in the reducing capacity of p53 mutant cells revealed by a short (1 h) exposure to a glycolytic inhibitor. Most importantly, this radiosensitisation of mutant p53 tumour cells by transient exposure to a glycolytic inhibitor should be therapeutically achievable, requiring a similar approach to that being used for the dosing of patients in the NIMRAD trial (NCT01950689) with nimorazole 90 min prior to irradiation [51]. We propose that future studies should investigate the combinatorial therapeutic potential of augmenting radiation therapy by administering a glycolytic inhibitor in a similar regime to that used for nimorazole prior to delivering each fraction of radiation in patients stratified by their *TP53* status.

Our data suggest that either p53 mutant cells produce more ROS in response to IR than p53 wild-type cells when glycolysis is inhibited or they are more sensitive to the ROS produced than wild-type cells (or a combination of the two). We therefore investigated the mechanism of this effect and found that in p53 mutant cells the addition of N-acetyl cysteine exogenously to the cells can overcome the increased radiation sensitivity in the presence of 2-DG as determined by either short-term or long term indicators (apoptosis and clonogenicity respectively). Thus we conclude that the increased sensitivity to IR in the presence of 2-DG is due to limiting availability of reducing agents such as glutathione that can reduce ROS. Although this effect could still be due to p53 mutant cells having greater intrinsic sensitivity to ROS, it seems simpler to propose that increased glycolytic flux leads to a shortage of reducing capacity. This would be expected in cells exhibiting high levels of glycolysis due to the high rate of conversion of glucose 6-phosphate into fructose 6–phosphate thus depleting the substrate for the oxidative branch of the pentose phosphate pathway [52].

It has been well-documented that loss of p53 function is generally linked with poorer patient outcomes in a wide range of common cancers including breast [1] and of particular significance here, in SCCHN [3,34,53]. Such a link immediately identifies p53 and more specifically *TP53* mutations as potential biomarkers either with prognostic utility as published or as we will argue here as potential predictive biomarkers. Most efforts to take advantage of *TP53* mutations for patient stratification envisage one of two scenarios. Firstly, in patients that retain wild-type p53 pharmacological means to increase the activity of p53 in tumour cells are conceived, such are the MDM2 and MDM4 antagonists. These were first developed in a credible form by Roche [54], but which now have been developed by numerous companies, some of which display a broader binding spectrum that includes both MDM2 and MDM4 [55]. Two problems can be anticipated with such approaches. One, that the strategy is limited, since it can be targeted only to patients likely to respond well to therapy since they retain p53 function [56]. Two, that applying a strong selective pressure to tumour cells that promotes selection for cells that harbour p53 mutations and thus to escape/resistance. The second scenario, focuses on patients that harbour mutations that inactivate p53. For tumours with mutations in the *TP53* gene, several strategies have been developed to exploit this. Two of the most common approaches aim either to reintroduce wild-type p53 or to rescue mutant p53 function. Delivering wild-type p53 generally depends upon gene targeting, and for now at least, no mechanism exists to achieve this in a tumour cell targeted or even somewhat targeted manner. Rescuing mutant p53 function, is prone to the same challenges as occur with other tumour suppressor proteins; there are many different mutations, and these affect the protein folding and function to differing degrees. Thus it is unlikely that any single agent could rescue the functions of diverse mutant proteins. Despite the overwhelming evidence that recovery of mutational loss of diverse tumour suppressor gene functions is potently tumour suppressive, such rescue is inherently difficult to achieve for any tumour suppressor except in a complex experimental system used for proof of principle [57]. One further strategy that deserves mention, is the development of viruses that display some selectivity for replication in *TP53* mutant cells and indeed, several have been developed that display such selectivity *in vitro*. However, whilst some beneficial responses have been observed in patients in clinical trials these were only observed in combination with other therapies, and moreover, it is not clear that p53 function is a determinant of *in vivo* biological effects [58].

Our data support the conclusion that a more promising strategy can be developed, that does not target p53 or mutations in *TP53*, but rather exploits cellular adaptations to loss of p53 function. Moreover, since cancer cells arise as a consequence of selection for adventitious mutations, it seems that targeting a single gene or protein merely provides a limited selective window, since escape from such selective pressures seems relatively likely. A more robust selection that targets a pathway adaptation, for example the metabolic adaptations studied here, must be more difficult to escape from, since other pressures have forced the tumour cells to adopt otherwise energetically lass favourable metabolic processes. Moreover, since such adaptations are common to the many and heterogeneous population of cells in the tumour, targeting such processes will affect the whole of the population. Warburg brilliantly anticipated this when he wrote in 1927: “The resistance of single tumor cells is not to be compared with that of single normal cells, but rather the tumor as a whole with the organism as a whole. An overpopulated city is more sensitive to stoppage of food supply than a normally populated city, even when the inhabitants can all endure hunger alike” [15]. Of course, with the metabolic adaptations that tumour cells display, these cells are inherently more sensitive to “starvation”. Thus the potential to exploit this adaptation for therapeutic effect seems particularly attractive and we argue that the studies presented here provide evidence of the direct functional link between p53 status and tumour metabolism in SCCHN cells that could be readily exploited by combining a glycolytic inhibitor with standard radiotherapy for patient benefit. Future experiments in a pre-clinical model are clearly needed and warranted to provide further evidence of proof of principle, and to develop and refine a therapeutic strategy of the kind envisaged as a result of the present studies.

An important aspect of these studies is that they demonstrate that loss of p53 function, whether by loss of protein expression (for example through a splice site mutation), missense mutation leading to variously loss of function only, dominant negative activity (DNA), gain of function (GOF) or through down-regulation of wild-type p53 using RNAi leads to a Warburg-type phenotype and more specifically to a loss of metabolic flexibility in SCCHN cells. This presents us with an opportunity for developing therapeutic interventions that focus on the metabolic changes, that is not restricted to a specific type of p53 mutation, nor is it rendered inappropriate in the presence of p53 GOF. Thus, these data suggest that such an approach could lead to a novel therapeutic strategy that should be widely applicable given the high prevalence for p53 mutations in SCCHN (CATG report ca. 80% in non-HPV disease [4]).

Most importantly, our results demonstrate that since *TP53* mutation or loss of function confers increased dependence on glycolysis and increased sensitivity to ROS with the resulting increased effectiveness of IR, the strategy identified provides for a more effective treatment approach for patients with mutations in *TP53*, precisely those that currently perform less well.

## CRediT authorship contribution statement

**Mark D. Wilkie:** Conceptualization, Investigation, Formal analysis, Writing - review & editing. **Emad A. Anaam:** Investigation. **Andrew S. Lau:** Investigation. **Carlos P. Rubbi:** Formal analysis, Writing - review & editing. **Terence M. Jones:** Conceptualization. **Mark T. Boyd:** Conceptualization, Funding acquisition, Supervision, Writing - original draft, Writing - review & editing. **Nikolina Vlatković:** Conceptualization, Methodology, Funding acquisition, Writing - original draft, Writing - review & editing.

## Declaration of competing interest

None of the authors have any conflicts of interest to declare.
