# Supplementary material for: TP53 mutations in head and neck cancer cells determine the Warburg phenotypic switch creating metabolic vulnerabilities and therapeutic opportunities for stratified therapies
Source: Cancer Lett. 2020 May 28;478:107–21. doi: 10.1016/j.canlet.2020.02.032 (PMC7133053; doi:10.1016/j.canlet.2020.02.032)
Supplement: Multimedia component 1 [file mmc1.pptx]

## Slide 1
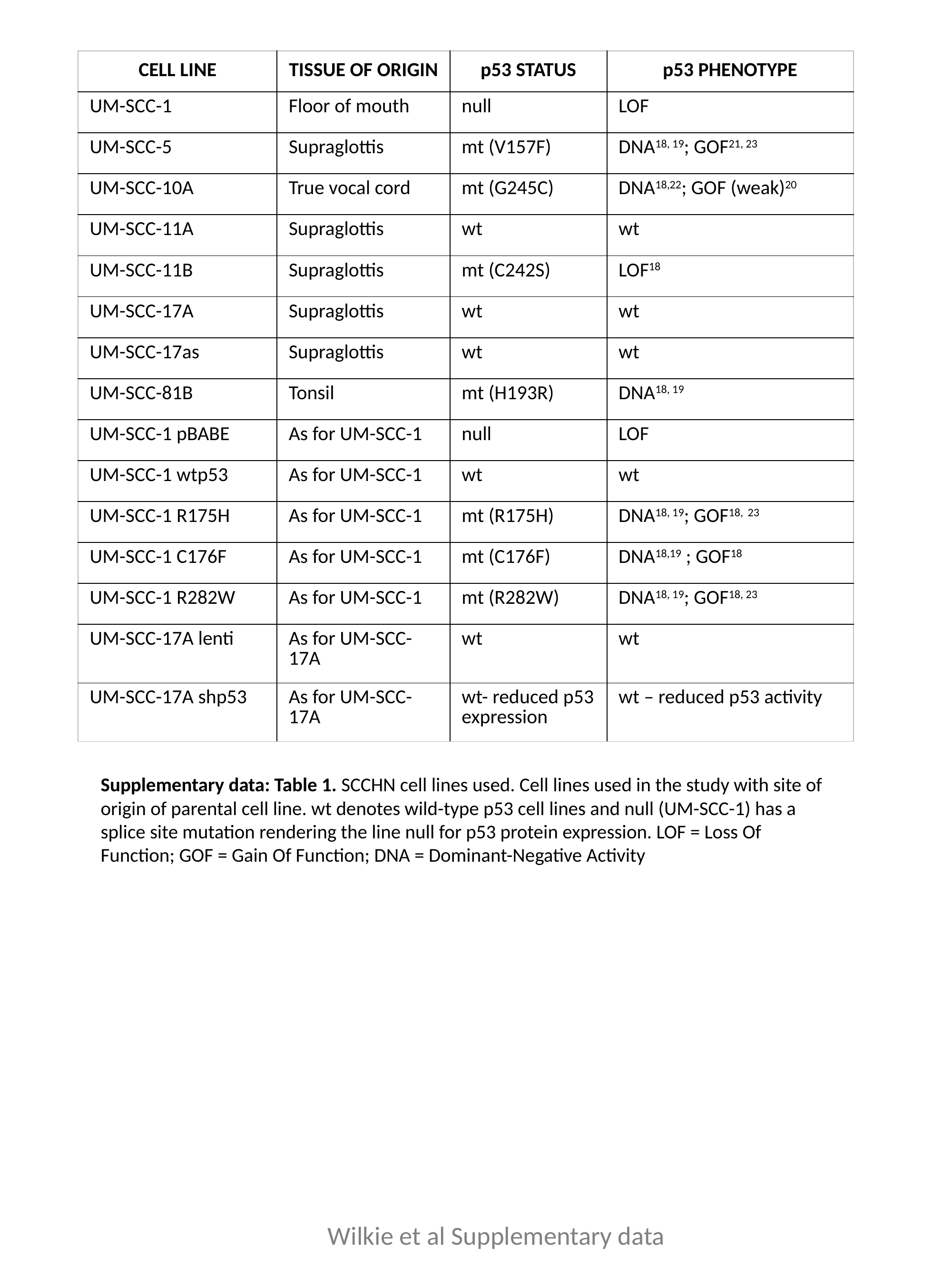

| CELL LINE | TISSUE OF ORIGIN | p53 STATUS | p53 PHENOTYPE |
| --- | --- | --- | --- |
| UM-SCC-1 | Floor of mouth | null | LOF |
| UM-SCC-5 | Supraglottis | mt (V157F) | DNA18, 19; GOF21, 23 |
| UM-SCC-10A | True vocal cord | mt (G245C) | DNA18,22; GOF (weak)20 |
| UM-SCC-11A | Supraglottis | wt | wt |
| UM-SCC-11B | Supraglottis | mt (C242S) | LOF18 |
| UM-SCC-17A | Supraglottis | wt | wt |
| UM-SCC-17as | Supraglottis | wt | wt |
| UM-SCC-81B | Tonsil | mt (H193R) | DNA18, 19 |
| UM-SCC-1 pBABE | As for UM-SCC-1 | null | LOF |
| UM-SCC-1 wtp53 | As for UM-SCC-1 | wt | wt |
| UM-SCC-1 R175H | As for UM-SCC-1 | mt (R175H) | DNA18, 19; GOF18, 23 |
| UM-SCC-1 C176F | As for UM-SCC-1 | mt (C176F) | DNA18,19 ; GOF18 |
| UM-SCC-1 R282W | As for UM-SCC-1 | mt (R282W) | DNA18, 19; GOF18, 23 |
| UM-SCC-17A lenti | As for UM-SCC-17A | wt | wt |
| UM-SCC-17A shp53 | As for UM-SCC-17A | wt- reduced p53 expression | wt – reduced p53 activity |
Supplementary data: Table 1. SCCHN cell lines used. Cell lines used in the study with site of origin of parental cell line. wt denotes wild-type p53 cell lines and null (UM-SCC-1) has a splice site mutation rendering the line null for p53 protein expression. LOF = Loss Of Function; GOF = Gain Of Function; DNA = Dominant-Negative Activity
Wilkie et al Supplementary data

## Slide 2
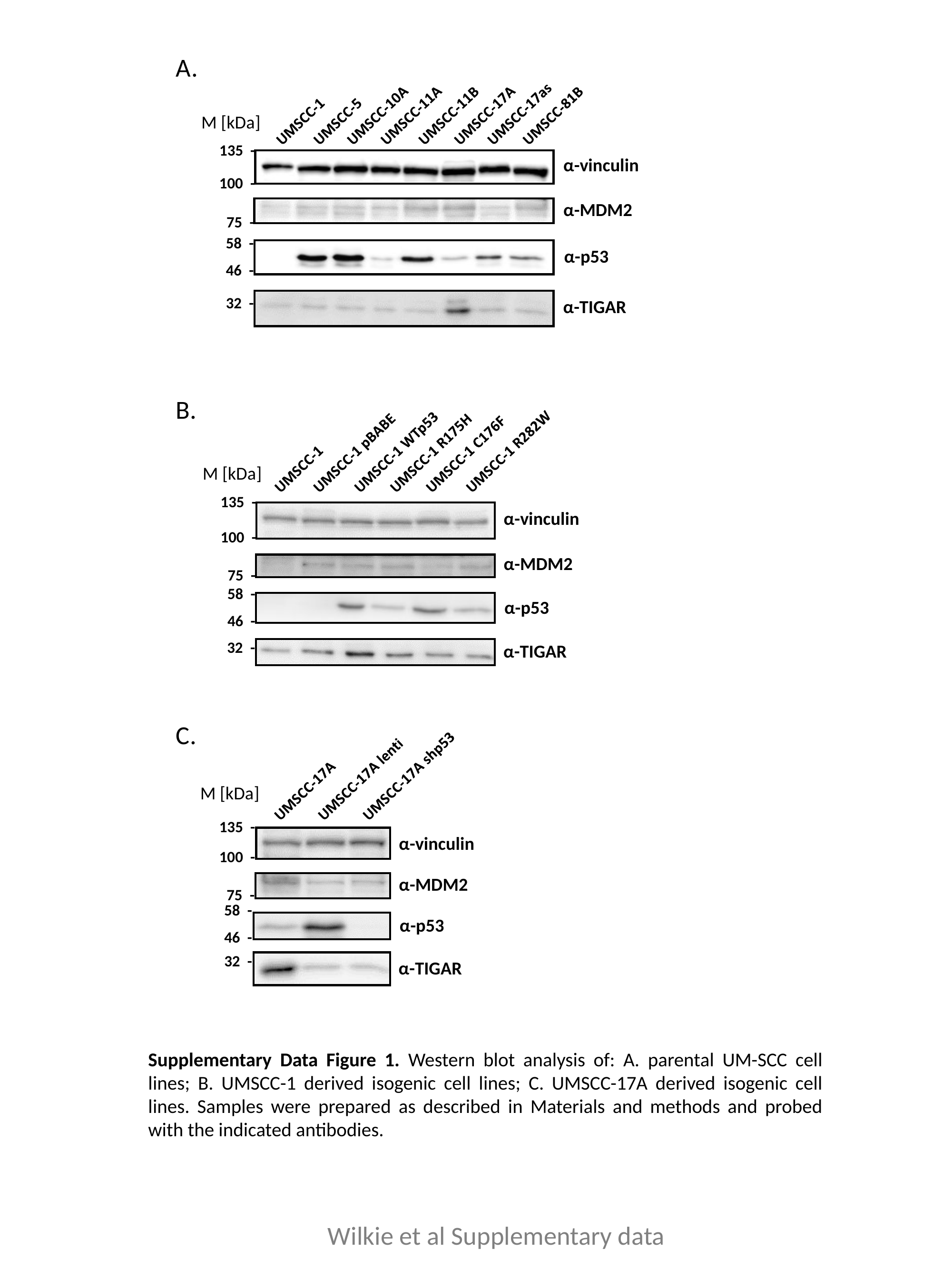

A.
B.
C.
Supplementary Data Figure 1. Western blot analysis of: A. parental UM-SCC cell lines; B. UMSCC-1 derived isogenic cell lines; C. UMSCC-17A derived isogenic cell lines. Samples were prepared as described in Materials and methods and probed with the indicated antibodies.
Wilkie et al Supplementary data

## Slide 3
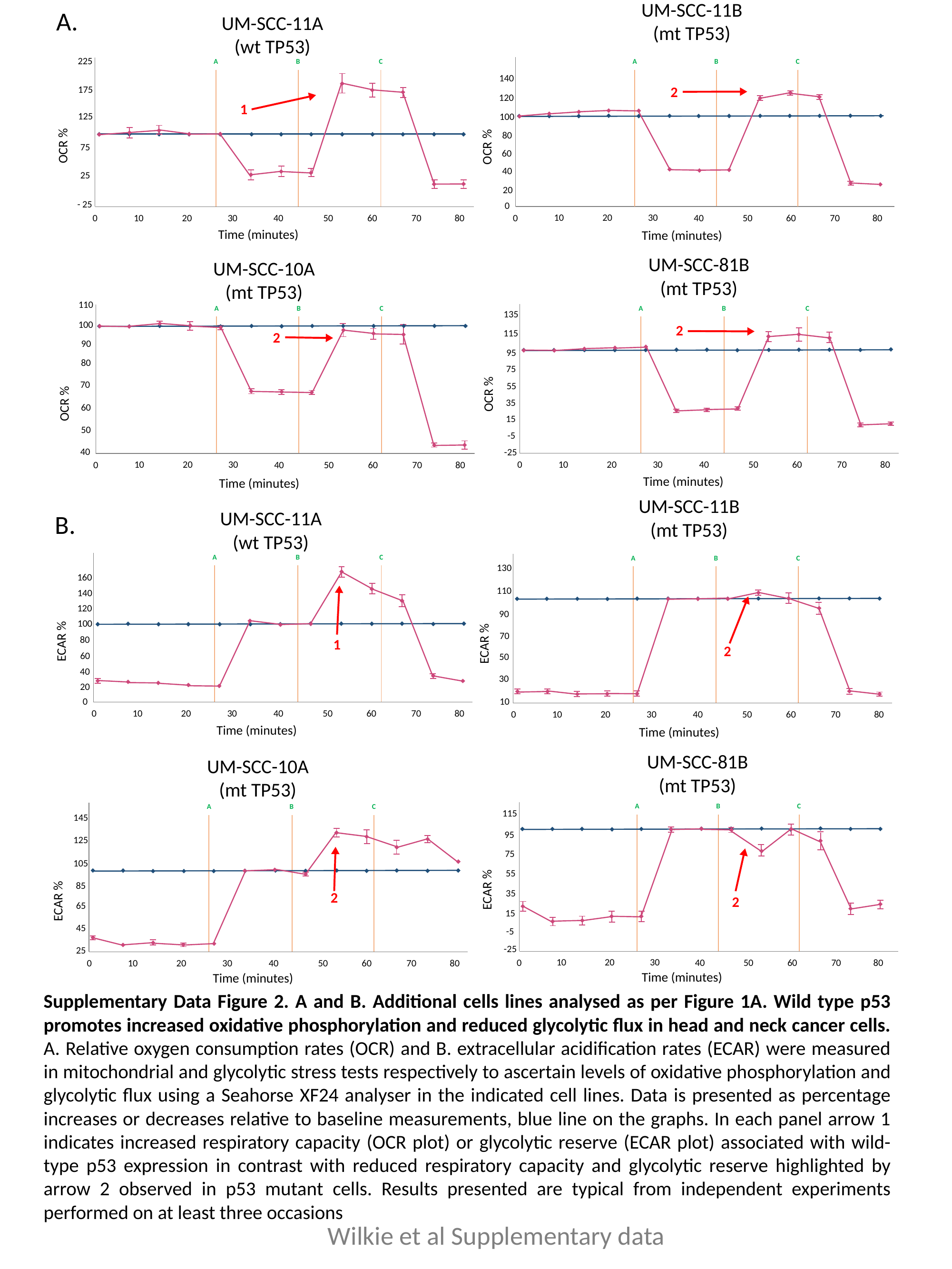

UM-SCC-11B
(mt TP53)
A.
UM-SCC-11A
(wt TP53)
OCR %
OCR %
Time (minutes)
Time (minutes)
UM-SCC-81B
(mt TP53)
UM-SCC-10A
(mt TP53)
OCR %
OCR %
Time (minutes)
Time (minutes)
UM-SCC-11B
(mt TP53)
ECAR %
Time (minutes)
UM-SCC-11A
(wt TP53)
ECAR %
Time (minutes)
B.
UM-SCC-81B
(mt TP53)
ECAR %
Time (minutes)
UM-SCC-10A
(mt TP53)
ECAR %
Time (minutes)
Supplementary Data Figure 2. A and B. Additional cells lines analysed as per Figure 1A. Wild type p53 promotes increased oxidative phosphorylation and reduced glycolytic flux in head and neck cancer cells. A. Relative oxygen consumption rates (OCR) and B. extracellular acidification rates (ECAR) were measured in mitochondrial and glycolytic stress tests respectively to ascertain levels of oxidative phosphorylation and glycolytic flux using a Seahorse XF24 analyser in the indicated cell lines. Data is presented as percentage increases or decreases relative to baseline measurements, blue line on the graphs. In each panel arrow 1 indicates increased respiratory capacity (OCR plot) or glycolytic reserve (ECAR plot) associated with wild-type p53 expression in contrast with reduced respiratory capacity and glycolytic reserve highlighted by arrow 2 observed in p53 mutant cells. Results presented are typical from independent experiments performed on at least three occasions
Wilkie et al Supplementary data

## Slide 4
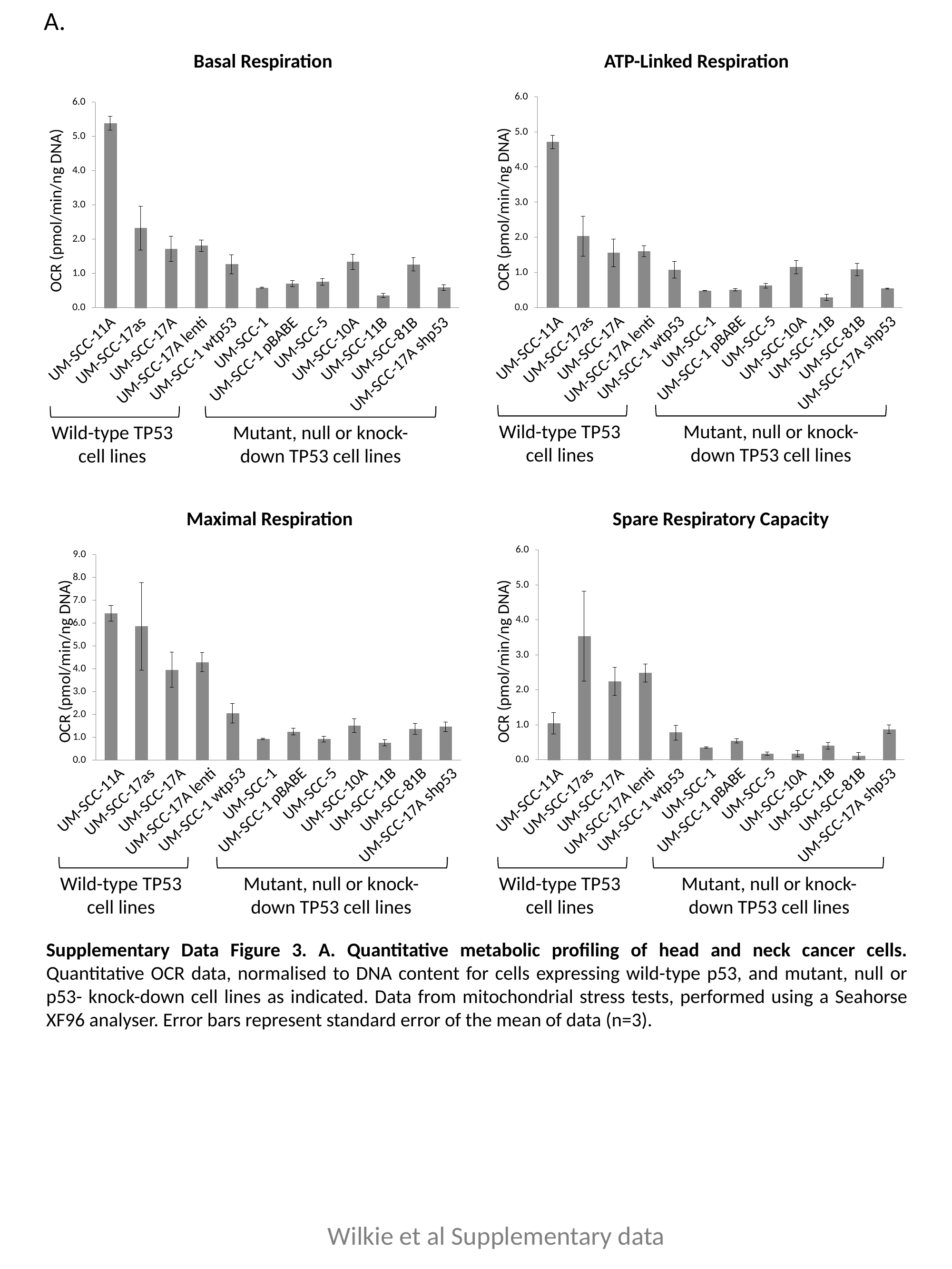

A.
Basal Respiration
ATP-Linked Respiration
OCR (pmol/min/ng DNA)
OCR (pmol/min/ng DNA)
UM-SCC-1
UM-SCC-5
UM-SCC-1
UM-SCC-5
UM-SCC-11B
UM-SCC-81B
UM-SCC-11A
UM-SCC-17A
UM-SCC-10A
UM-SCC-11B
UM-SCC-81B
UM-SCC-11A
UM-SCC-17A
UM-SCC-10A
UM-SCC-17as
UM-SCC-17as
UM-SCC-1 wtp53
UM-SCC-1 pBABE
UM-SCC-1 wtp53
UM-SCC-1 pBABE
UM-SCC-17A lenti
UM-SCC-17A lenti
UM-SCC-17A shp53
UM-SCC-17A shp53
Wild-type TP53 cell lines
Mutant, null or knock-down TP53 cell lines
Wild-type TP53 cell lines
Mutant, null or knock-down TP53 cell lines
Maximal Respiration
Spare Respiratory Capacity
OCR (pmol/min/ng DNA)
OCR (pmol/min/ng DNA)
UM-SCC-1
UM-SCC-5
UM-SCC-1
UM-SCC-5
UM-SCC-11B
UM-SCC-81B
UM-SCC-11B
UM-SCC-81B
UM-SCC-11A
UM-SCC-17A
UM-SCC-10A
UM-SCC-11A
UM-SCC-17A
UM-SCC-10A
UM-SCC-17as
UM-SCC-17as
UM-SCC-1 wtp53
UM-SCC-1 wtp53
UM-SCC-1 pBABE
UM-SCC-1 pBABE
UM-SCC-17A lenti
UM-SCC-17A lenti
UM-SCC-17A shp53
UM-SCC-17A shp53
Wild-type TP53 cell lines
Mutant, null or knock-down TP53 cell lines
Wild-type TP53 cell lines
Mutant, null or knock-down TP53 cell lines
Supplementary Data Figure 3. A. Quantitative metabolic profiling of head and neck cancer cells. Quantitative OCR data, normalised to DNA content for cells expressing wild-type p53, and mutant, null or p53- knock-down cell lines as indicated. Data from mitochondrial stress tests, performed using a Seahorse XF96 analyser. Error bars represent standard error of the mean of data (n=3).
Wilkie et al Supplementary data

## Slide 5
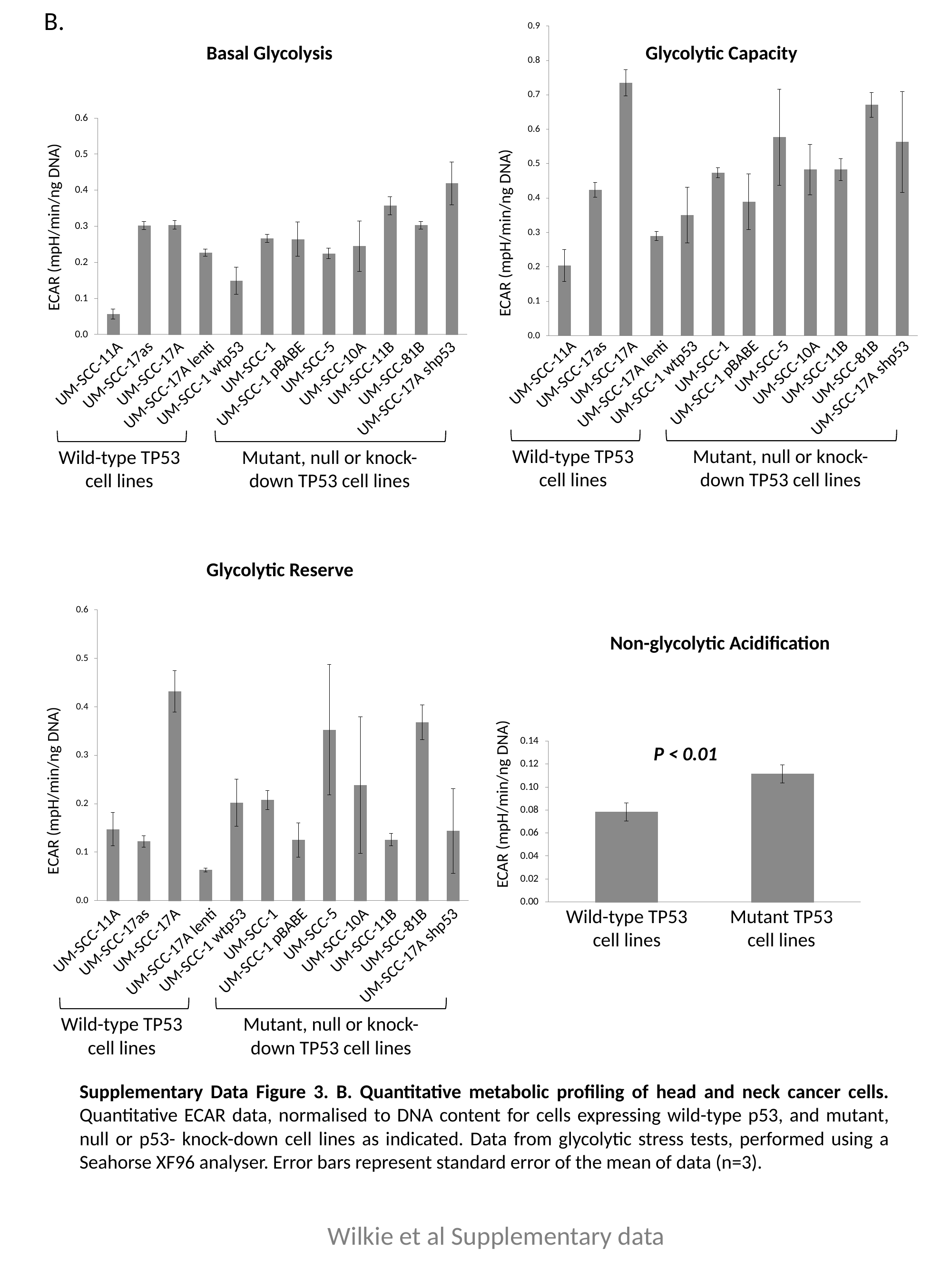

B.
Basal Glycolysis
Glycolytic Capacity
ECAR (mpH/min/ng DNA)
ECAR (mpH/min/ng DNA)
UM-SCC-1
UM-SCC-5
UM-SCC-1
UM-SCC-5
UM-SCC-11B
UM-SCC-81B
UM-SCC-11A
UM-SCC-17A
UM-SCC-10A
UM-SCC-11B
UM-SCC-81B
UM-SCC-11A
UM-SCC-17A
UM-SCC-10A
UM-SCC-17as
UM-SCC-17as
UM-SCC-1 wtp53
UM-SCC-1 pBABE
UM-SCC-1 wtp53
UM-SCC-1 pBABE
UM-SCC-17A lenti
UM-SCC-17A lenti
UM-SCC-17A shp53
UM-SCC-17A shp53
Wild-type TP53 cell lines
Mutant, null or knock-down TP53 cell lines
Wild-type TP53 cell lines
Mutant, null or knock-down TP53 cell lines
Glycolytic Reserve
Non-glycolytic Acidification
P < 0.01
ECAR (mpH/min/ng DNA)
ECAR (mpH/min/ng DNA)
Mutant TP53 cell lines
Wild-type TP53 cell lines
UM-SCC-1
UM-SCC-5
UM-SCC-11B
UM-SCC-81B
UM-SCC-11A
UM-SCC-17A
UM-SCC-10A
UM-SCC-17as
UM-SCC-1 wtp53
UM-SCC-1 pBABE
UM-SCC-17A lenti
UM-SCC-17A shp53
Wild-type TP53 cell lines
Mutant, null or knock-down TP53 cell lines
Supplementary Data Figure 3. B. Quantitative metabolic profiling of head and neck cancer cells. Quantitative ECAR data, normalised to DNA content for cells expressing wild-type p53, and mutant, null or p53- knock-down cell lines as indicated. Data from glycolytic stress tests, performed using a Seahorse XF96 analyser. Error bars represent standard error of the mean of data (n=3).
Wilkie et al Supplementary data

## Slide 6
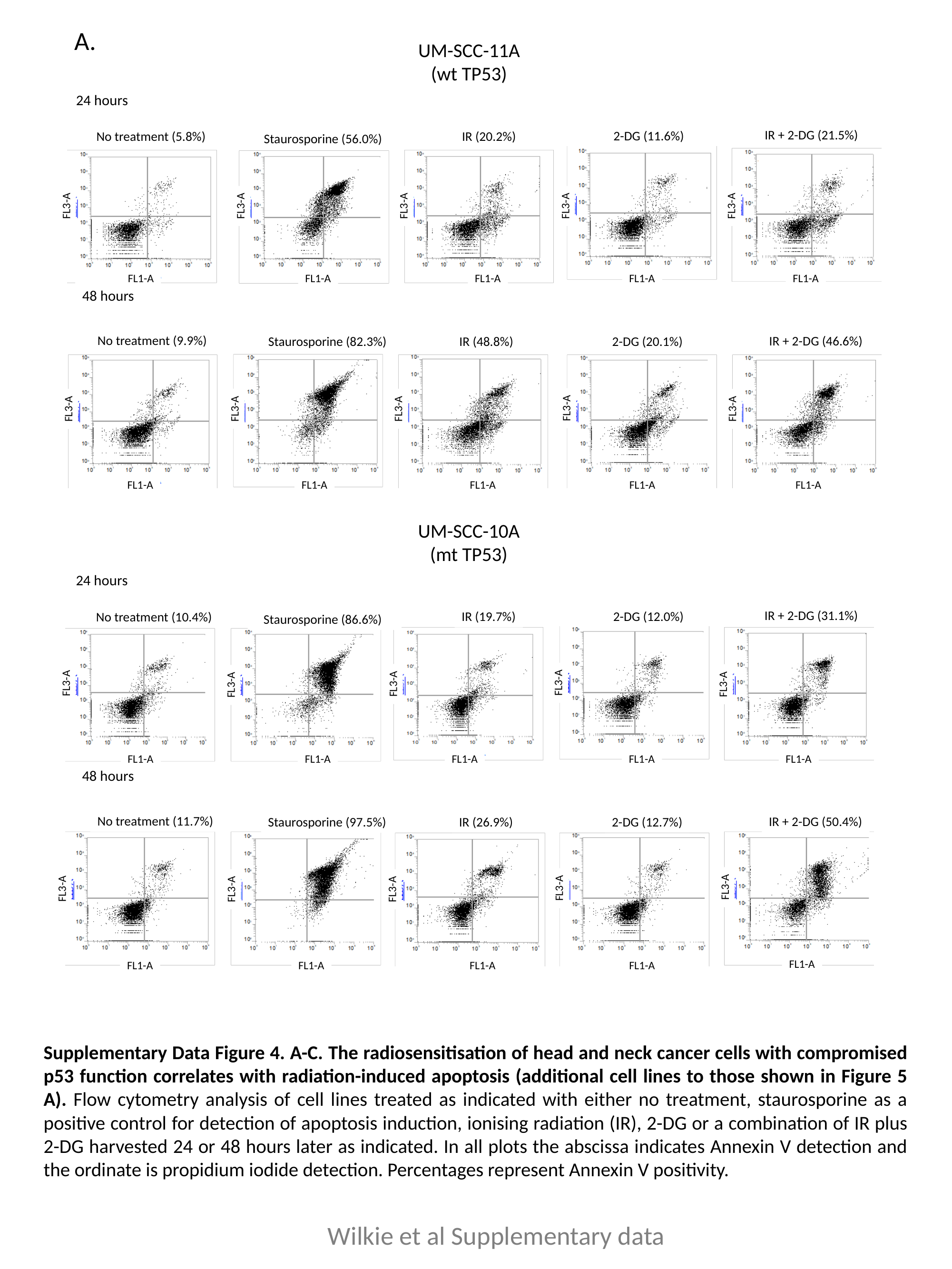

A.
UM-SCC-11A
(wt TP53)
24 hours
IR + 2-DG (21.5%)
2-DG (11.6%)
IR (20.2%)
No treatment (5.8%)
 Staurosporine (56.0%)
FL3-A
FL3-A
FL3-A
FL3-A
FL3-A
FL1-A
FL1-A
FL1-A
FL1-A
FL1-A
48 hours
No treatment (9.9%)
IR + 2-DG (46.6%)
IR (48.8%)
Staurosporine (82.3%)
2-DG (20.1%)
FL3-A
FL3-A
FL3-A
FL3-A
FL3-A
FL1-A
FL1-A
FL1-A
FL1-A
FL1-A
UM-SCC-10A
(mt TP53)
24 hours
IR + 2-DG (31.1%)
2-DG (12.0%)
IR (19.7%)
No treatment (10.4%)
 Staurosporine (86.6%)
FL3-A
FL3-A
FL3-A
FL3-A
FL3-A
FL1-A
FL1-A
FL1-A
FL1-A
FL1-A
48 hours
No treatment (11.7%)
IR + 2-DG (50.4%)
IR (26.9%)
Staurosporine (97.5%)
2-DG (12.7%)
FL3-A
FL3-A
FL3-A
FL3-A
FL3-A
FL1-A
FL1-A
FL1-A
FL1-A
FL1-A
Supplementary Data Figure 4. A-C. The radiosensitisation of head and neck cancer cells with compromised p53 function correlates with radiation-induced apoptosis (additional cell lines to those shown in Figure 5 A). Flow cytometry analysis of cell lines treated as indicated with either no treatment, staurosporine as a positive control for detection of apoptosis induction, ionising radiation (IR), 2-DG or a combination of IR plus 2-DG harvested 24 or 48 hours later as indicated. In all plots the abscissa indicates Annexin V detection and the ordinate is propidium iodide detection. Percentages represent Annexin V positivity.
Wilkie et al Supplementary data

## Slide 7
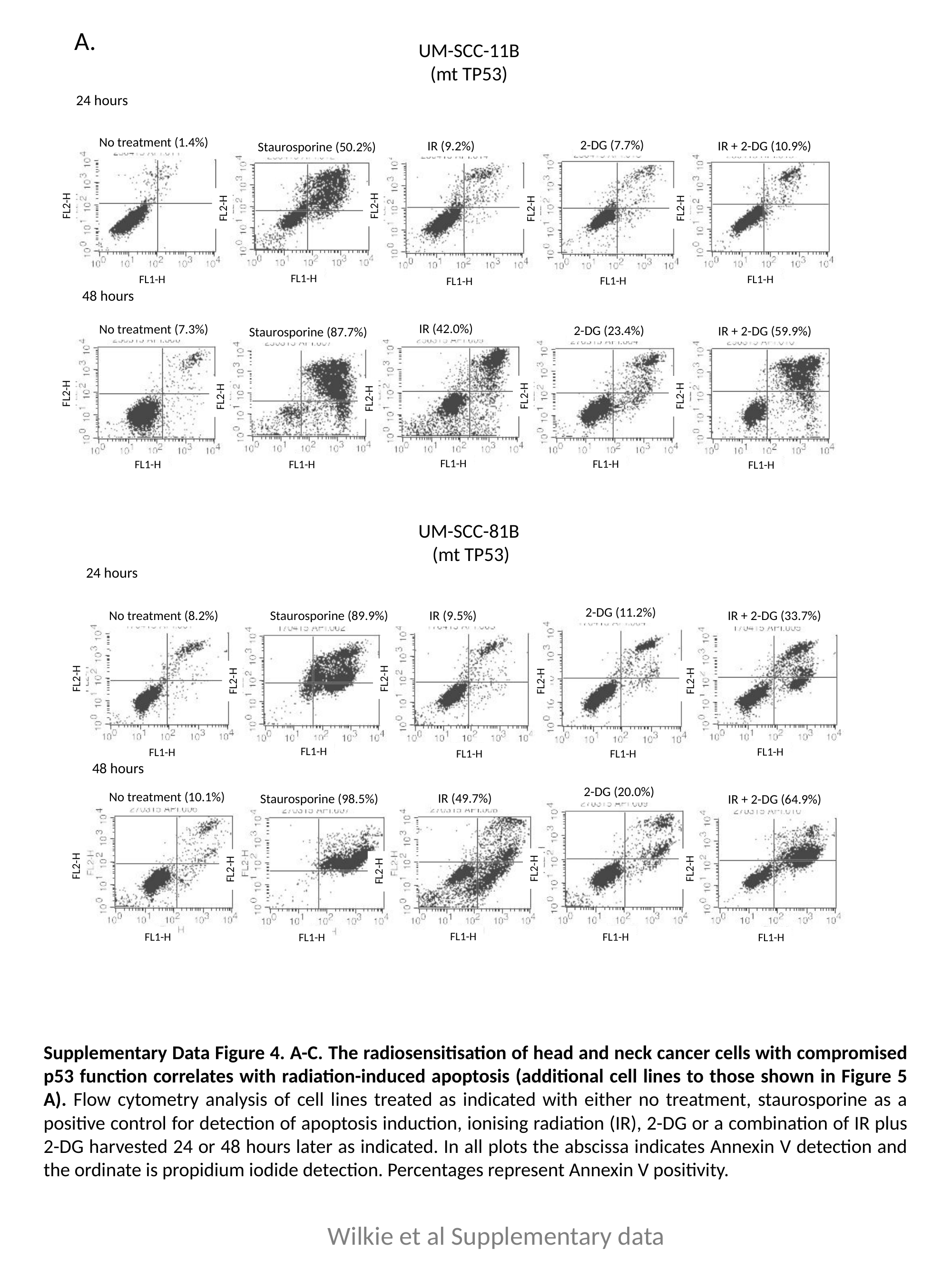

A.
UM-SCC-11B
(mt TP53)
24 hours
No treatment (1.4%)
2-DG (7.7%)
IR (9.2%)
IR + 2-DG (10.9%)
 Staurosporine (50.2%)
FL2-H
FL2-H
FL2-H
FL2-H
FL2-H
FL1-H
FL1-H
FL1-H
FL1-H
FL1-H
48 hours
IR (42.0%)
No treatment (7.3%)
2-DG (23.4%)
IR + 2-DG (59.9%)
Staurosporine (87.7%)
FL2-H
FL2-H
FL2-H
FL2-H
FL2-H
FL1-H
FL1-H
FL1-H
FL1-H
FL1-H
UM-SCC-81B
 (mt TP53)
24 hours
2-DG (11.2%)
No treatment (8.2%)
IR (9.5%)
IR + 2-DG (33.7%)
 Staurosporine (89.9%)
FL2-H
FL2-H
FL2-H
FL2-H
FL2-H
FL1-H
FL1-H
FL1-H
FL1-H
FL1-H
48 hours
2-DG (20.0%)
No treatment (10.1%)
IR (49.7%)
Staurosporine (98.5%)
IR + 2-DG (64.9%)
FL2-H
FL2-H
FL2-H
FL2-H
FL2-H
FL1-H
FL1-H
FL1-H
FL1-H
FL1-H
Supplementary Data Figure 4. A-C. The radiosensitisation of head and neck cancer cells with compromised p53 function correlates with radiation-induced apoptosis (additional cell lines to those shown in Figure 5 A). Flow cytometry analysis of cell lines treated as indicated with either no treatment, staurosporine as a positive control for detection of apoptosis induction, ionising radiation (IR), 2-DG or a combination of IR plus 2-DG harvested 24 or 48 hours later as indicated. In all plots the abscissa indicates Annexin V detection and the ordinate is propidium iodide detection. Percentages represent Annexin V positivity.
Wilkie et al Supplementary data

## Slide 8
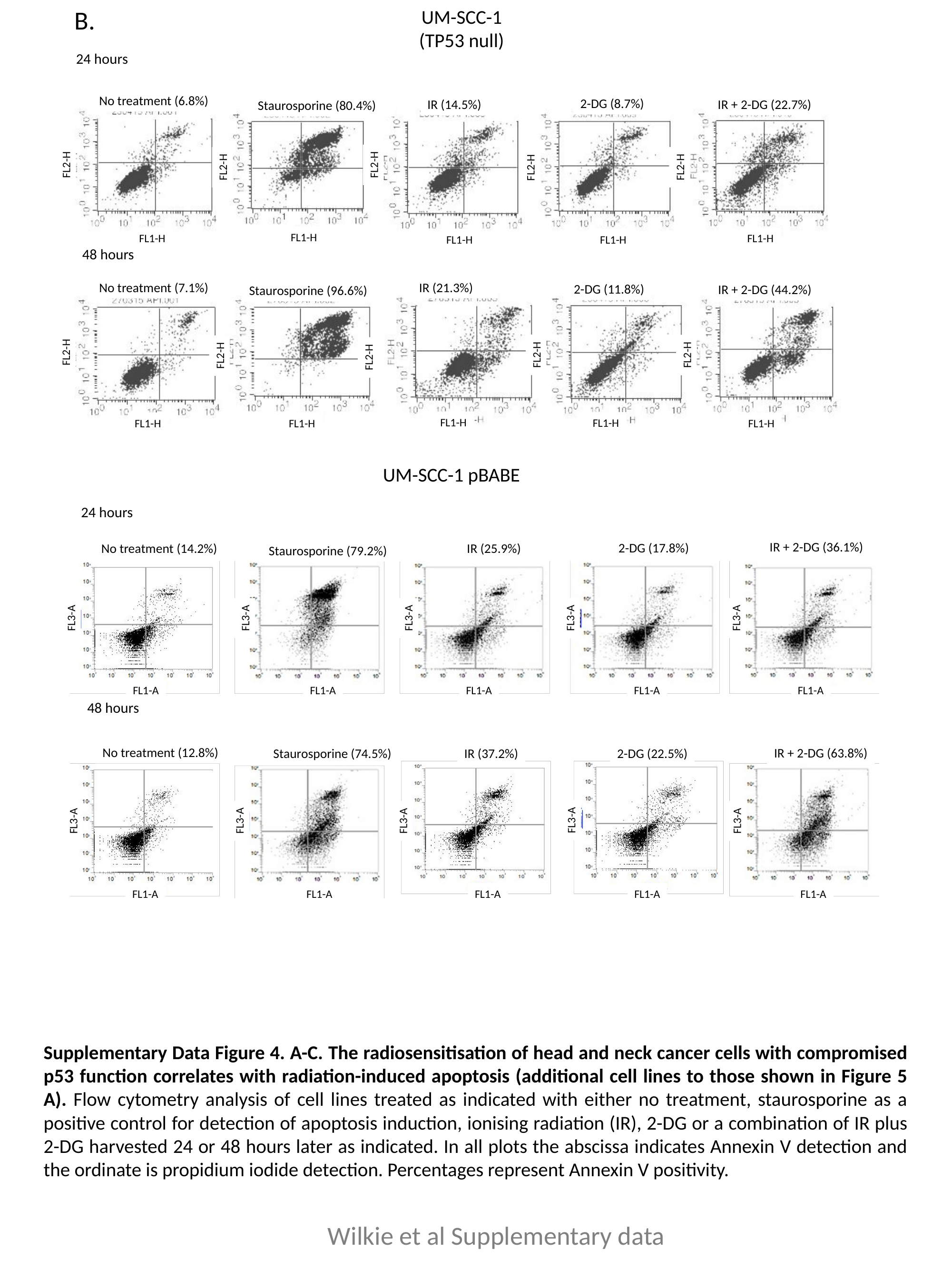

B.
UM-SCC-1
(TP53 null)
24 hours
No treatment (6.8%)
2-DG (8.7%)
IR (14.5%)
IR + 2-DG (22.7%)
 Staurosporine (80.4%)
FL2-H
FL2-H
FL2-H
FL2-H
FL2-H
FL1-H
FL1-H
FL1-H
FL1-H
FL1-H
48 hours
IR (21.3%)
No treatment (7.1%)
2-DG (11.8%)
IR + 2-DG (44.2%)
Staurosporine (96.6%)
FL2-H
FL2-H
FL2-H
FL2-H
FL2-H
FL1-H
FL1-H
FL1-H
FL1-H
FL1-H
UM-SCC-1 pBABE
24 hours
IR + 2-DG (36.1%)
2-DG (17.8%)
IR (25.9%)
No treatment (14.2%)
 Staurosporine (79.2%)
FL3-A
FL3-A
FL3-A
FL3-A
FL3-A
FL1-A
FL1-A
FL1-A
FL1-A
FL1-A
48 hours
No treatment (12.8%)
IR + 2-DG (63.8%)
IR (37.2%)
Staurosporine (74.5%)
2-DG (22.5%)
FL3-A
FL3-A
FL3-A
FL3-A
FL3-A
FL1-A
FL1-A
FL1-A
FL1-A
FL1-A
Supplementary Data Figure 4. A-C. The radiosensitisation of head and neck cancer cells with compromised p53 function correlates with radiation-induced apoptosis (additional cell lines to those shown in Figure 5 A). Flow cytometry analysis of cell lines treated as indicated with either no treatment, staurosporine as a positive control for detection of apoptosis induction, ionising radiation (IR), 2-DG or a combination of IR plus 2-DG harvested 24 or 48 hours later as indicated. In all plots the abscissa indicates Annexin V detection and the ordinate is propidium iodide detection. Percentages represent Annexin V positivity.
Wilkie et al Supplementary data

## Slide 9
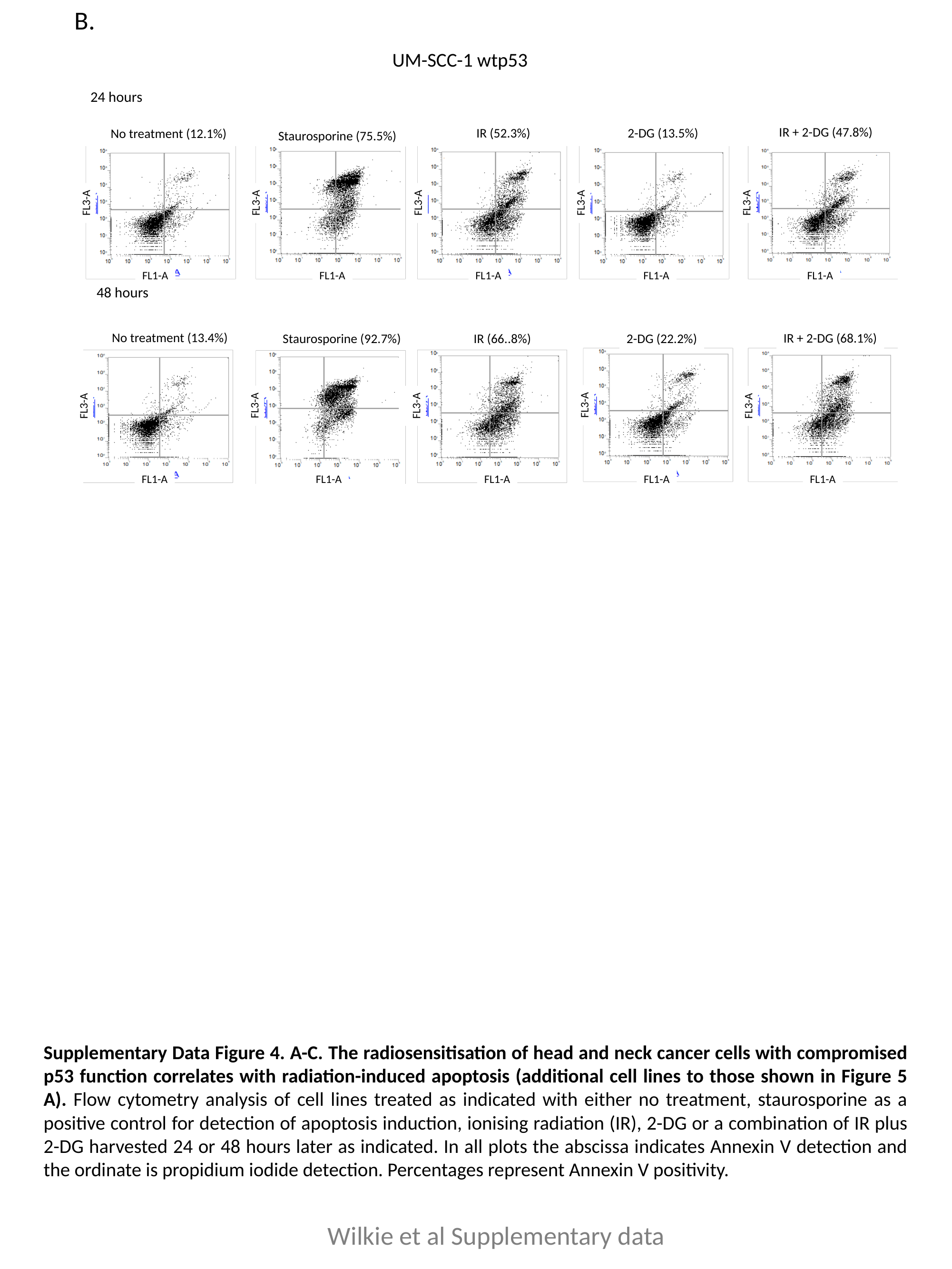

B.
UM-SCC-1 wtp53
24 hours
IR + 2-DG (47.8%)
2-DG (13.5%)
IR (52.3%)
No treatment (12.1%)
 Staurosporine (75.5%)
FL3-A
FL3-A
FL3-A
FL3-A
FL3-A
FL1-A
FL1-A
FL1-A
FL1-A
FL1-A
48 hours
No treatment (13.4%)
IR + 2-DG (68.1%)
IR (66..8%)
Staurosporine (92.7%)
2-DG (22.2%)
FL3-A
FL3-A
FL3-A
FL3-A
FL3-A
FL1-A
FL1-A
FL1-A
FL1-A
FL1-A
Supplementary Data Figure 4. A-C. The radiosensitisation of head and neck cancer cells with compromised p53 function correlates with radiation-induced apoptosis (additional cell lines to those shown in Figure 5 A). Flow cytometry analysis of cell lines treated as indicated with either no treatment, staurosporine as a positive control for detection of apoptosis induction, ionising radiation (IR), 2-DG or a combination of IR plus 2-DG harvested 24 or 48 hours later as indicated. In all plots the abscissa indicates Annexin V detection and the ordinate is propidium iodide detection. Percentages represent Annexin V positivity.
Wilkie et al Supplementary data

## Slide 10
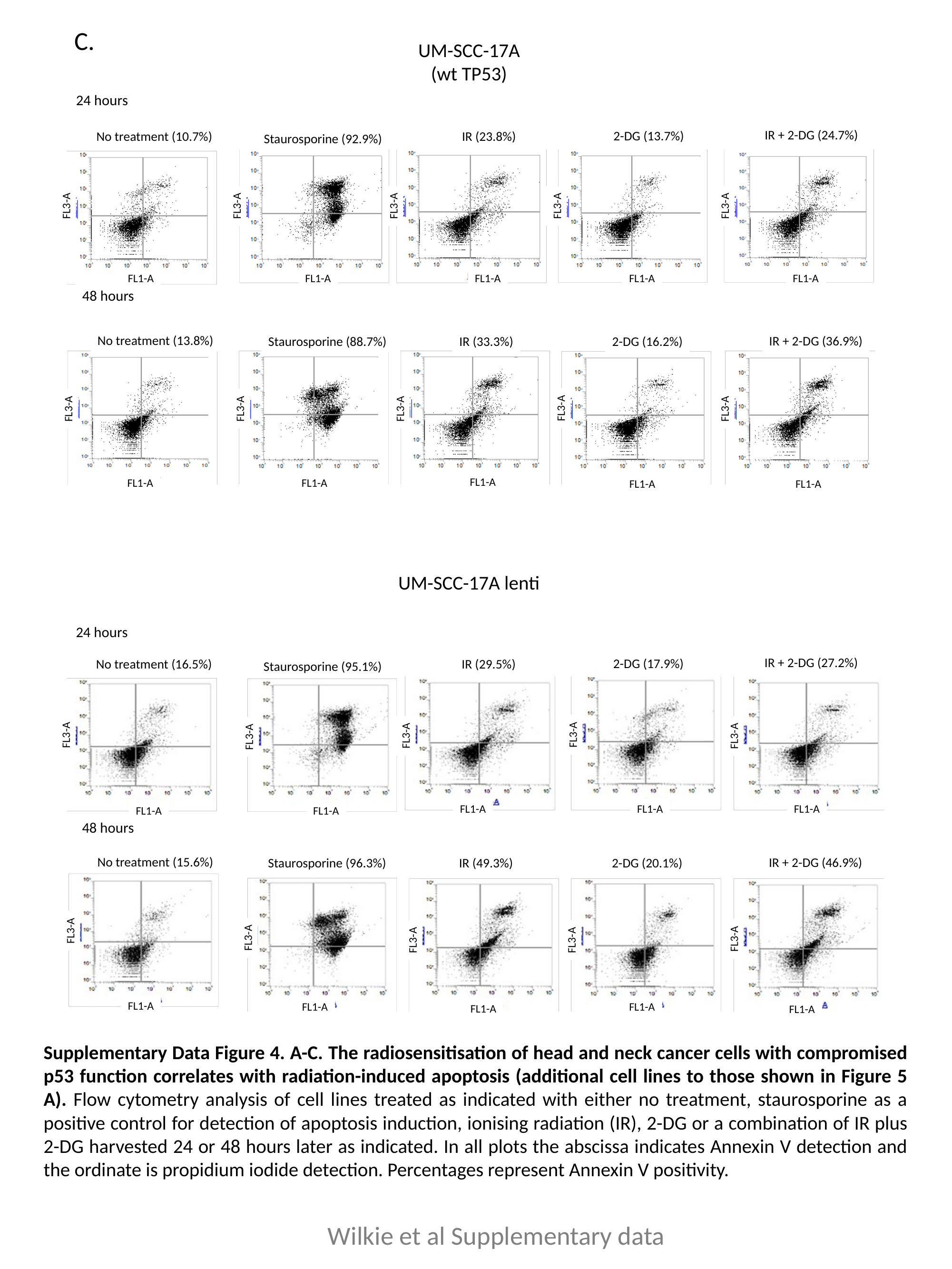

C.
UM-SCC-17A
(wt TP53)
24 hours
IR + 2-DG (24.7%)
2-DG (13.7%)
IR (23.8%)
No treatment (10.7%)
 Staurosporine (92.9%)
FL3-A
FL3-A
FL3-A
FL3-A
FL3-A
FL1-A
FL1-A
FL1-A
FL1-A
FL1-A
48 hours
No treatment (13.8%)
IR + 2-DG (36.9%)
IR (33.3%)
Staurosporine (88.7%)
2-DG (16.2%)
FL3-A
FL3-A
FL3-A
FL3-A
FL3-A
FL1-A
FL1-A
FL1-A
FL1-A
FL1-A
UM-SCC-17A lenti
24 hours
IR + 2-DG (27.2%)
2-DG (17.9%)
IR (29.5%)
No treatment (16.5%)
 Staurosporine (95.1%)
FL3-A
FL3-A
FL3-A
FL3-A
FL3-A
FL1-A
FL1-A
FL1-A
FL1-A
FL1-A
48 hours
No treatment (15.6%)
IR + 2-DG (46.9%)
IR (49.3%)
Staurosporine (96.3%)
2-DG (20.1%)
FL3-A
FL3-A
FL3-A
FL3-A
FL3-A
FL1-A
FL1-A
FL1-A
FL1-A
FL1-A
Supplementary Data Figure 4. A-C. The radiosensitisation of head and neck cancer cells with compromised p53 function correlates with radiation-induced apoptosis (additional cell lines to those shown in Figure 5 A). Flow cytometry analysis of cell lines treated as indicated with either no treatment, staurosporine as a positive control for detection of apoptosis induction, ionising radiation (IR), 2-DG or a combination of IR plus 2-DG harvested 24 or 48 hours later as indicated. In all plots the abscissa indicates Annexin V detection and the ordinate is propidium iodide detection. Percentages represent Annexin V positivity.
Wilkie et al Supplementary data

## Slide 11
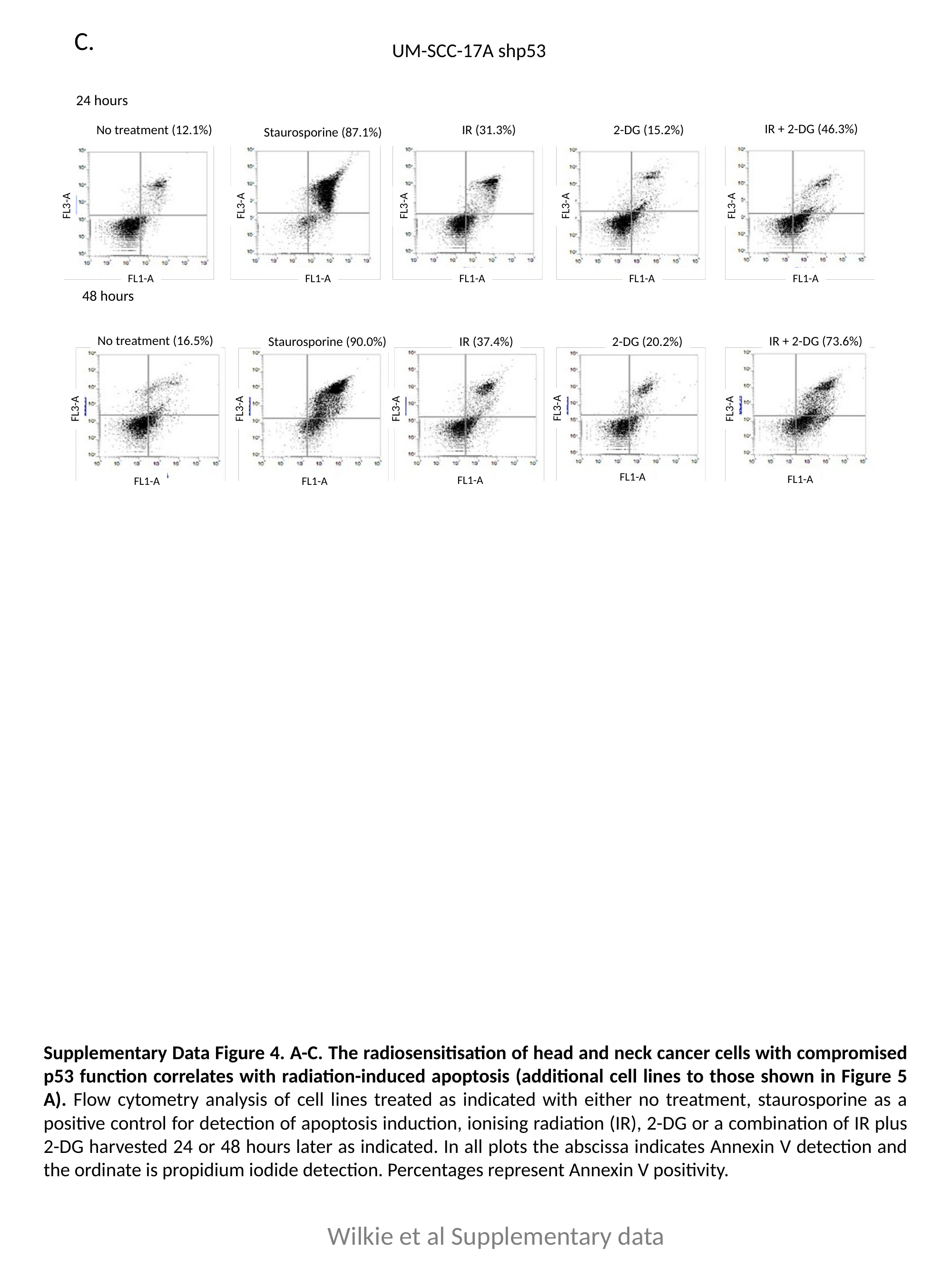

C.
UM-SCC-17A shp53
24 hours
IR + 2-DG (46.3%)
2-DG (15.2%)
IR (31.3%)
No treatment (12.1%)
 Staurosporine (87.1%)
FL3-A
FL3-A
FL3-A
FL3-A
FL3-A
FL1-A
FL1-A
FL1-A
FL1-A
FL1-A
48 hours
No treatment (16.5%)
IR + 2-DG (73.6%)
IR (37.4%)
Staurosporine (90.0%)
2-DG (20.2%)
FL3-A
FL3-A
FL3-A
FL3-A
FL3-A
FL1-A
FL1-A
FL1-A
FL1-A
FL1-A
Supplementary Data Figure 4. A-C. The radiosensitisation of head and neck cancer cells with compromised p53 function correlates with radiation-induced apoptosis (additional cell lines to those shown in Figure 5 A). Flow cytometry analysis of cell lines treated as indicated with either no treatment, staurosporine as a positive control for detection of apoptosis induction, ionising radiation (IR), 2-DG or a combination of IR plus 2-DG harvested 24 or 48 hours later as indicated. In all plots the abscissa indicates Annexin V detection and the ordinate is propidium iodide detection. Percentages represent Annexin V positivity.
Wilkie et al Supplementary data

## Slide 12
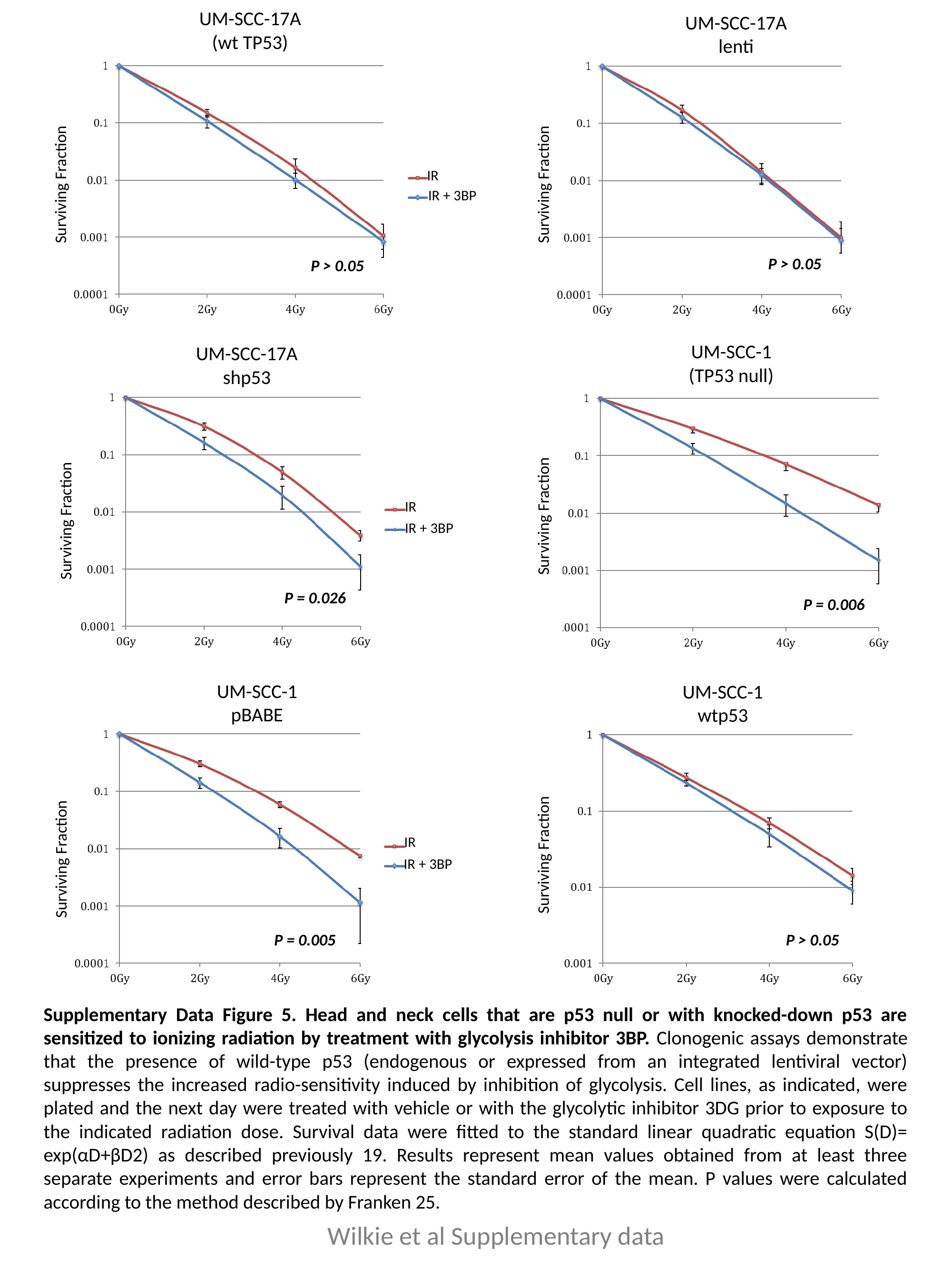

UM-SCC-17A
(wt TP53)
UM-SCC-17A
lenti
IR
Surviving Fraction
Surviving Fraction
IR + 3BP
P > 0.05
P > 0.05
UM-SCC-1
(TP53 null)
UM-SCC-17A
shp53
IR
Surviving Fraction
Surviving Fraction
IR + 3BP
P = 0.026
P = 0.006
UM-SCC-1
pBABE
UM-SCC-1
wtp53
IR
Surviving Fraction
Surviving Fraction
IR + 3BP
P = 0.005
P > 0.05
Supplementary Data Figure 5. Head and neck cells that are p53 null or with knocked-down p53 are sensitized to ionizing radiation by treatment with glycolysis inhibitor 3BP. Clonogenic assays demonstrate that the presence of wild-type p53 (endogenous or expressed from an integrated lentiviral vector) suppresses the increased radio-sensitivity induced by inhibition of glycolysis. Cell lines, as indicated, were plated and the next day were treated with vehicle or with the glycolytic inhibitor 3DG prior to exposure to the indicated radiation dose. Survival data were fitted to the standard linear quadratic equation S(D)= exp(αD+βD2) as described previously 19. Results represent mean values obtained from at least three separate experiments and error bars represent the standard error of the mean. P values were calculated according to the method described by Franken 25.
Wilkie et al Supplementary data

## Slide 13
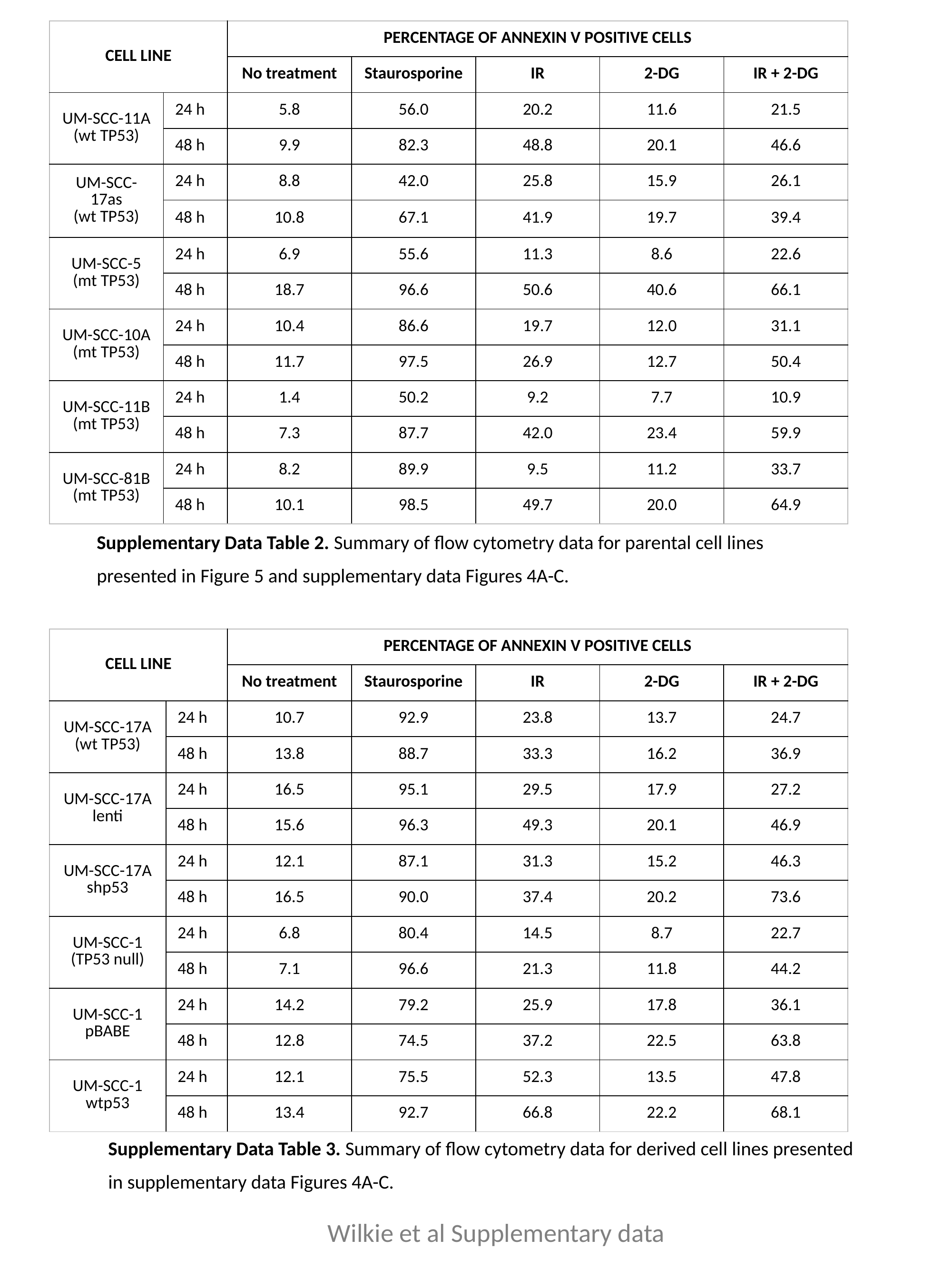

| CELL LINE | | PERCENTAGE OF ANNEXIN V POSITIVE CELLS | | | | |
| --- | --- | --- | --- | --- | --- | --- |
| | | No treatment | Staurosporine | IR | 2-DG | IR + 2-DG |
| UM-SCC-11A (wt TP53) | 24 h | 5.8 | 56.0 | 20.2 | 11.6 | 21.5 |
| | 48 h | 9.9 | 82.3 | 48.8 | 20.1 | 46.6 |
| UM-SCC-17as (wt TP53) | 24 h | 8.8 | 42.0 | 25.8 | 15.9 | 26.1 |
| | 48 h | 10.8 | 67.1 | 41.9 | 19.7 | 39.4 |
| UM-SCC-5 (mt TP53) | 24 h | 6.9 | 55.6 | 11.3 | 8.6 | 22.6 |
| | 48 h | 18.7 | 96.6 | 50.6 | 40.6 | 66.1 |
| UM-SCC-10A (mt TP53) | 24 h | 10.4 | 86.6 | 19.7 | 12.0 | 31.1 |
| | 48 h | 11.7 | 97.5 | 26.9 | 12.7 | 50.4 |
| UM-SCC-11B (mt TP53) | 24 h | 1.4 | 50.2 | 9.2 | 7.7 | 10.9 |
| | 48 h | 7.3 | 87.7 | 42.0 | 23.4 | 59.9 |
| UM-SCC-81B (mt TP53) | 24 h | 8.2 | 89.9 | 9.5 | 11.2 | 33.7 |
| | 48 h | 10.1 | 98.5 | 49.7 | 20.0 | 64.9 |
Supplementary Data Table 2. Summary of flow cytometry data for parental cell lines presented in Figure 5 and supplementary data Figures 4A-C.
| CELL LINE | | PERCENTAGE OF ANNEXIN V POSITIVE CELLS | | | | |
| --- | --- | --- | --- | --- | --- | --- |
| | | No treatment | Staurosporine | IR | 2-DG | IR + 2-DG |
| UM-SCC-17A (wt TP53) | 24 h | 10.7 | 92.9 | 23.8 | 13.7 | 24.7 |
| | 48 h | 13.8 | 88.7 | 33.3 | 16.2 | 36.9 |
| UM-SCC-17A lenti | 24 h | 16.5 | 95.1 | 29.5 | 17.9 | 27.2 |
| | 48 h | 15.6 | 96.3 | 49.3 | 20.1 | 46.9 |
| UM-SCC-17A shp53 | 24 h | 12.1 | 87.1 | 31.3 | 15.2 | 46.3 |
| | 48 h | 16.5 | 90.0 | 37.4 | 20.2 | 73.6 |
| UM-SCC-1 (TP53 null) | 24 h | 6.8 | 80.4 | 14.5 | 8.7 | 22.7 |
| | 48 h | 7.1 | 96.6 | 21.3 | 11.8 | 44.2 |
| UM-SCC-1 pBABE | 24 h | 14.2 | 79.2 | 25.9 | 17.8 | 36.1 |
| | 48 h | 12.8 | 74.5 | 37.2 | 22.5 | 63.8 |
| UM-SCC-1 wtp53 | 24 h | 12.1 | 75.5 | 52.3 | 13.5 | 47.8 |
| | 48 h | 13.4 | 92.7 | 66.8 | 22.2 | 68.1 |
Supplementary Data Table 3. Summary of flow cytometry data for derived cell lines presented in supplementary data Figures 4A-C.
Wilkie et al Supplementary data

## Slide 14
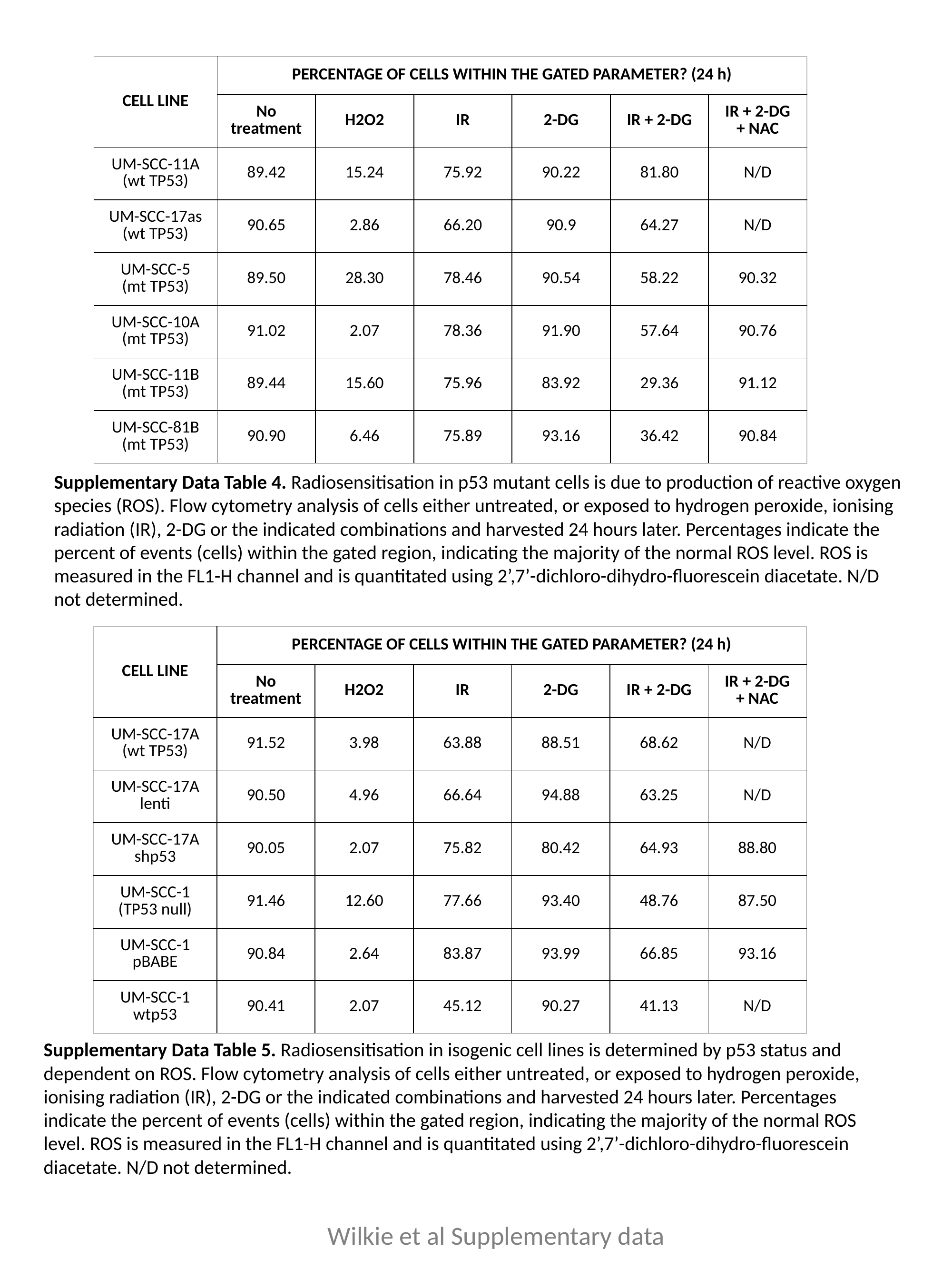

| CELL LINE | PERCENTAGE OF CELLS WITHIN THE GATED PARAMETER? (24 h) | | | | | |
| --- | --- | --- | --- | --- | --- | --- |
| | No treatment | H2O2 | IR | 2-DG | IR + 2-DG | IR + 2-DG + NAC |
| UM-SCC-11A (wt TP53) | 89.42 | 15.24 | 75.92 | 90.22 | 81.80 | N/D |
| UM-SCC-17as (wt TP53) | 90.65 | 2.86 | 66.20 | 90.9 | 64.27 | N/D |
| UM-SCC-5 (mt TP53) | 89.50 | 28.30 | 78.46 | 90.54 | 58.22 | 90.32 |
| UM-SCC-10A (mt TP53) | 91.02 | 2.07 | 78.36 | 91.90 | 57.64 | 90.76 |
| UM-SCC-11B (mt TP53) | 89.44 | 15.60 | 75.96 | 83.92 | 29.36 | 91.12 |
| UM-SCC-81B (mt TP53) | 90.90 | 6.46 | 75.89 | 93.16 | 36.42 | 90.84 |
Supplementary Data Table 4. Radiosensitisation in p53 mutant cells is due to production of reactive oxygen species (ROS). Flow cytometry analysis of cells either untreated, or exposed to hydrogen peroxide, ionising radiation (IR), 2-DG or the indicated combinations and harvested 24 hours later. Percentages indicate the percent of events (cells) within the gated region, indicating the majority of the normal ROS level. ROS is measured in the FL1-H channel and is quantitated using 2’,7’-dichloro-dihydro-fluorescein diacetate. N/D not determined.
| CELL LINE | PERCENTAGE OF CELLS WITHIN THE GATED PARAMETER? (24 h) | | | | | |
| --- | --- | --- | --- | --- | --- | --- |
| | No treatment | H2O2 | IR | 2-DG | IR + 2-DG | IR + 2-DG + NAC |
| UM-SCC-17A (wt TP53) | 91.52 | 3.98 | 63.88 | 88.51 | 68.62 | N/D |
| UM-SCC-17A lenti | 90.50 | 4.96 | 66.64 | 94.88 | 63.25 | N/D |
| UM-SCC-17A shp53 | 90.05 | 2.07 | 75.82 | 80.42 | 64.93 | 88.80 |
| UM-SCC-1 (TP53 null) | 91.46 | 12.60 | 77.66 | 93.40 | 48.76 | 87.50 |
| UM-SCC-1 pBABE | 90.84 | 2.64 | 83.87 | 93.99 | 66.85 | 93.16 |
| UM-SCC-1 wtp53 | 90.41 | 2.07 | 45.12 | 90.27 | 41.13 | N/D |
Supplementary Data Table 5. Radiosensitisation in isogenic cell lines is determined by p53 status and dependent on ROS. Flow cytometry analysis of cells either untreated, or exposed to hydrogen peroxide, ionising radiation (IR), 2-DG or the indicated combinations and harvested 24 hours later. Percentages indicate the percent of events (cells) within the gated region, indicating the majority of the normal ROS level. ROS is measured in the FL1-H channel and is quantitated using 2’,7’-dichloro-dihydro-fluorescein diacetate. N/D not determined.
Wilkie et al Supplementary data
